# Evaluation of the effect of CYP2D6 and OCT1 polymorphisms on the pharmacokinetics of tramadol: Implications for clinical safety and dose rationale in paediatric chronic pain

**DOI:** 10.1111/bcp.16201

**Published:** 2024-10-09

**Authors:** Paul Healy, Karel Allegaert, Oscar Della Pasqua

**Affiliations:** ^1^ Clinical Pharmacology & Therapeutics Group University College London London UK; ^2^ Department of Development and Regeneration KU Leuven Leuven Belgium; ^3^ Department of Pharmaceutical and Pharmacological Sciences KU Leuven Leuven Belgium; ^4^ Department of Hospital Pharmacy Erasmus MC Rotterdam The Netherlands; ^5^ Clinical Pharmacology Modelling and Simulation GlaxoSmithKline London UK

**Keywords:** chronic pain, dose rationale, extrapolation, CYP2D6, OCT1, paediatrics, tramadol

## Abstract

**Aims:**

Our investigation aimed to assess the dose rationale of tramadol in paediatric patients considering the effect of CYP2D6/OCT1 polymorphisms on systemic exposure. Recommendations were made for the oral dose of tramadol to be used in a prospective study in children (3 months to < 18 years old) with chronic pain.

**Methods:**

Intravenous pharmacokinetic and genotype data from neonatal patients (*n* = 46) were available for this analysis. The time course of tramadol and O‐desmethyltramadol (M1) concentrations was characterized using a nonlinear mixed effects approach in conjunction with extrapolation principles. Clinical trial simulations were then implemented to explore the effects of polymorphism, maturation and developmental growth on the disposition of tramadol and M1. Reported efficacious exposure range in adult subjects were used as reference.

**Results:**

The pharmacokinetics of tramadol and M1 was characterized by a two‐compartment model. The total clearance of tramadol (CLPP) comprised CYP2D6‐mediated metabolism (CLPM) and other pathways (CLPO). Age‐related changes in CLPM, CLPO and M1 clearance (CLMO) were described by a sigmoid function, with CYP2D6 as a covariate on CLPP and CLPM,  and OCT1 on CLMO. Simulation scenarios including different CYP2D6/OCT1 combinations revealed that steady‐state concentrations are above the putative ranges for analgesia in >15% and >70% of subjects after doses of 3 and 8 mg/kg, respectively.

**Conclusions:**

In the absence of genotyping, reference exposure ranges can be used to define the dose rationale for tramadol in paediatric chronic pain. However, a starting dose of 0.5 mg/kg/day should be considered, followed by stepwise titration to the desired analgesic response.

**What is already known about this subject**

- Tramadol is prescribed to adults for acute and chronic pain treatment, but it is also used off‐label in young children.
- Large interindividual variation in the pharmacokinetics of tramadol, including CYP2D6 and OCT1 genetic polymorphism, makes it challenging to establish a simplified dose rationale for children.
- Tramadol efficacy in children may be extrapolated from adults and adolescents based on the assumption of a comparable concentration–effect relationship across populations.

**What this study adds**

- CYP2D6 and OCT1 polymorphisms are associated with clinically relevant increases in systemic exposure to tramadol and its active metabolite (O‐desmethyltramadol).
- Given the contribution of genetic and non‐genetic (maturational) factors to interindividual variability in drug exposure, titration to response is recommended to ensure adequate analgesia and acceptable safety profile in the target paediatric population.
- Whilst the benefit–risk balance should be carefully considered when prescribing opioids for paediatric patients, a starting dose of 0.5 mg/kg/day tramadol minimizes the risks associated with potentially high exposure to O‐desmethyltramadol in subjects with concurrent CYP2D6 and OCT1 variants.

## 1 INTRODUCTION

In adults, the synthetic opioid tramadol is approved for use in moderate to severe nociceptive pain (acute and chronic), usually in combination with non‐opioid analgesics.^1^ As an analgesic, tramadol has one‐tenth of the potency of morphine but carries a better side‐effect profile with fewer incidences of respiratory depression, nausea and vomiting, constipation and sedation.^2^ Notwithstanding the warnings and restrictions issued by regulators in the European Union (EU) and in the United States,^3^, ^4^ codeine and tramadol are still prescribed for treating moderate to severe nociceptive pain in children. In fact, the approval of tramadol for paediatric use as well as its off‐label use in children varies across countries. In the United States, tramadol is approved only in adults,^5^ whereas in the EU, after initial approval in the paediatric population above 1–3 years of age, tramadol use was restricted to adolescents >12 years old.^6^ Despite this context and the lack of consensus on alternative treatment choices and dosing regimens, its off‐label use remains significant.

Even though a definitive recommendation for the paediatric dosing of tramadol supported by a clinical study based on pharmacokinetic‐pharmacodynamic (PKPD) principles is not yet available, it can be anticipated that empirical evidence from such a clinical trial may not allow characterization of the effect of intrinsic factors (i.e., pharmacokinetic covariates) on the overall interindividual variability in the exposure to tramadol and its metabolite, O‐desmethyltramadol [M1]. The formation of this metabolite is of great clinical relevance, as it has been shown that M1 has a six‐fold higher potency relative to the parent drug with >200 times greater affinity for the μ‐opioid receptors.^1^, ^7^

Tramadol exerts its effects by inhibiting the neuronal uptake of serotonin and norepinephrine.^8^ However, an integrated analysis of the exposure to both moieties is required to assess the efficacy and safety profile of tramadol. In fact, lowering of the dose of tramadol during the first days of treatment is an important factor in improving tolerability,^9^, ^10^ i.e., a consequence of the reduction in circulating levels of M1. Nevertheless, tramadol use is not without safety concerns. A notable case report recounts severe respiratory depression in a boy aged 5 years who underwent a tonsillectomy and was prescribed 200 mg tramadol for postoperative pain relief. The patient presented with lethargy, pin‐point pupils, respiratory depression and oxygen saturation of 48% in room air. Genotyping of cytochrome P450 (CYP)2D6 was conducted, finding three functional alleles that were consistent with ultra‐rapid metabolism (UM), which led to highly increased plasma concentrations of M1.^11^ This incident has been flagged by the Food and Drug Administration (FDA), issuing a boxed warning in 2015 regarding the risks of use (off‐label) of tramadol in children <12 years of age.^12^

Polymorphisms of genes encoding CYP2D6 may result in wide interindividual variation in drug metabolism and, therefore, in M1 plasma concentrations, which contribute substantially to the pharmacodynamic properties of tramadol. Individuals with more than two active copies of CYP2D6 are classified as ultra‐rapid metabolizers (UM)^13^ and more likely to show an increased incidence of adverse effects. Conversely, poor efficacy can be seen among poor metabolizers (PM), i.e. those individuals having no active copies of the CYP2D6 gene. In PM patients, tramadol treatment has been found to provide little or no pain relief via M1.^13^ Indeed, a study in postoperative pain in adults showed PMs demanded higher tramadol doses compared with individuals with extensive (“normal”) metabolic activity (EM).^14^

In addition to the polymorphism in metabolite formation, a study by Matic et al.^15^ has highlighted the effects of genetic variation in transporters, and more specifically heritable loss of organic cation transporter (OCT)1 activity. The role of OCT1 in the pharmacokinetics of M1 may be explained by the rate‐limiting activity of OCT1 in the reuptake of M1 into the hepatocytes as the initial step of M1 inactivation. Individuals with reduced or absent OCT1 activity show higher circulating blood concentrations of M1 and, consequently, increased analgesic activity.^16^, ^17^ As reported, if M1 is not taken up by the hepatocytes, it will remain in the circulation and will not be metabolized and cleared effectively.

Based on the aforementioned, it becomes evident that interindividual differences in exposure and response to tramadol will depend not only on the known demographic covariate effects (e.g., age, weight) on drug disposition but also on CYP2D6 and OCT1 polymorphism. The purpose of this investigation was therefore to characterize the implications of the concurrent effects of developmental growth, including maturational and genetic covariate factors on the disposition of tramadol and M1. Using population modelling and simulation techniques, a model‐based evaluation of the effect of CYP2D6 and OCT1 polymorphism is proposed for the optimization and potential personalization of the dosing regimen of tramadol for the treatment of paediatric patients with chronic pain. The results from this investigation aimed at supporting the dose rationale for a clinical study in children from 3 months to <18 years old, in which the safety and efficacy of tramadol is compared with gabapentin (GABA‐1: NCT02722603).^18^

## 2 METHODS

**FIGURE 1:**
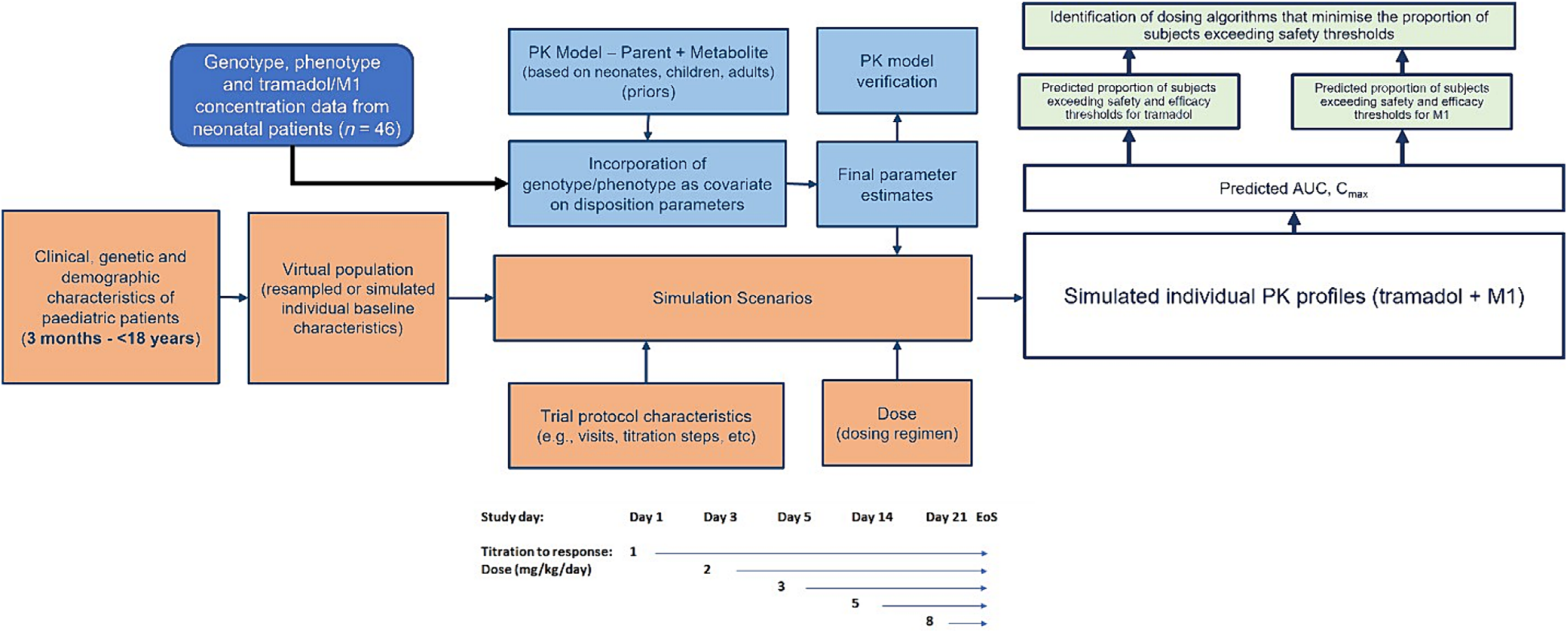
Flow diagram describing the steps required for the optimization of tramadol dosing regimen in paediatric patients taking into account genetic and non‐genetic factors associated with developmental growth and maturation processes known to affect drug disposition. Simulation scenarios were based on a clinical trial protocol including up to five titration steps. EoS, end of study; PK, pharmacokinetic.

The steps associated with model implementation and evaluation, as well as the simulation of relevant clinical scenarios aimed at exploring dose optimization and predicting the safety profile in the paediatric population are summarized in Figure 1; details on each of the steps are described in the subsequent sections.

### 2.1 Clinical data

The demographic and pharmacokinetic (PK) data used to perform this analysis were obtained from a set of clinical studies including neonates.^19^, ^20^ Out of the original study population (*n* = 122), 52 patients were neonates and had genotype information, but data from only 46 patients of this group were suitable for the purpose of this study; genotyping was not available for the remaining population, which consisted of older children and adults. Our final study population included male and female patients with median postmenstrual age (PMA) of 40 weeks and body weight of 3.2 kg. Tramadol was administered intravenously with an average loading dose of 2.1 mg/kg over 30 min, followed by a continuous infusion of 0.35 mg/kg/h. Full demographic details of the clinical data are summarized in Table 1. In total, 349 blood samples were available for tramadol and 229 for M1, with the majority of patients having plasma concentration measurements up to a duration of 24 h post‐infusion of tramadol. Approval for the study protocol was granted by the local ethics board of the University Hospital, Leuven, Belgium. Additional details on the clinical study protocols can be found elsewhere.^19^, ^20^

**TABLE 1:** Overview of the baseline characteristics of the patient population (*n* = 46) available for the development of a joint model describing the pharmacokinetics of tramadol and its metabolite M1.

Baseline characteristics	
Median PMA in weeks (range)	40 (31–54)
Median weight in kg (range)	3.2 (0.93–6.14)
Sex (male/female)	32/14
CYP2D6 activity score	*3* = 2, *2* = 29, *1* = 15
OCT1 activity score	*0* = 2, *1* = 20, *2* = 24

Abbreviation: PMA, postmenstrual age.

Patient exclusion (*n* = 6) was due to the very limited sampling points available for the prediction of individual PK profiles with sufficient precision or outlying concentration values, which when omitted singularly still caused termination of the minimization. Overall, there were 247 measured tramadol concentrations and 223 concentrations for M1 in the final analysis data set. Drug concentrations were obtained by means of gas chromatography with nitrogen‐selective detection. Full details on the bioanalysis of both moieties can be found elsewhere.^21^, ^22^ In brief, bioanalytical methods showed linearity within the concentration range of 2.5–500 ng/mL. The precision and accuracy of the assay were high with a coefficient of variation (CV%) of 4.2–8.4% for tramadol and 2.9–8.4% for M1, with a lower detection limit of 2.0 ng/mL for tramadol and 2.5 ng/mL for M1.

### 2.2 Magnitude of the effect of polymorphism (phenotype) and pharmacokinetic targets for analgesia

To accurately characterize the clinical implications of genetic variation on the systemic exposure to tramadol and its M1 metabolite, detailed information on the prevalence of each genotype is required (upper panel of Table 2). These data need to be linked to the corresponding phenotype, which together with other covariates contribute or determine interindividual differences in drug disposition and systemic exposure. Of interest is the combination of CYP2D6 and OCT1 activity scores, which results in exposure to tramadol and M1 exceeding the anticipated safety thresholds, as these individuals are likely to experience adverse events (lower panel of Table 2). Therefore, a trigger for overexposure was set when steady‐state plasma concentrations reach 10% and 5% over the putative therapeutic range, for tramadol and M1, respectively. A steady state plasma concentration range of 200–300 ng/mL for tramadol has been identified as therapeutic target for pain relief. Steady‐state concentrations between 33 and 50 ng/mL were considered as therapeutic target for the metabolite (M1). These ranges are well below the safety threshold for adverse events.^26^

**TABLE 2:** Upper panel: prevalence of the different phenotypes based on CYP2D6/OCT1 activity scores (AS)^23^ and corresponding groups or categories used for the purpose of the current study. Lower panel: prevalence of concurrent polymorphism in CYP2D6 and OCT1.^17^, ^24^

CYP2D6 activity score (AS)	Population prevalence	Phenotypical grouping used as discrete covariates in this study
Poor (AS = 0)	0.4–5.4%	**G1** (AS = 0–0.5)
Intermediate (AS = 0.5)	0.4–11%	
Extensive (AS = 1)	67–90%	**G2** (AS = 1–1.5)
Extensive (AS = 1.5)		
Extensive (AS = 2)		**G3** (AS = 2–3)
Ultra Rapid (AS >2)	1–21%	

OCT1	Population prevalence	
0 active	9%	**G0**
1 active	38%	**G1**
2 active	53%	**G2**

CYP2D6 activity score	OCT1 activity score	Population prevalence
3	0	0.5%
2	1	‐
1	2	‐
3	1	‐
2	2	‐
1	0	‐
3	2	‐
2	0	3.6%
1	1	‐

It is worth noting that the low numbers of PMs in our study (Table 1) reflects the prevalence of this group in the general population (Table 2).

### 2.3 Pharmacokinetic modelling and extrapolation

**FIGURE 2:**
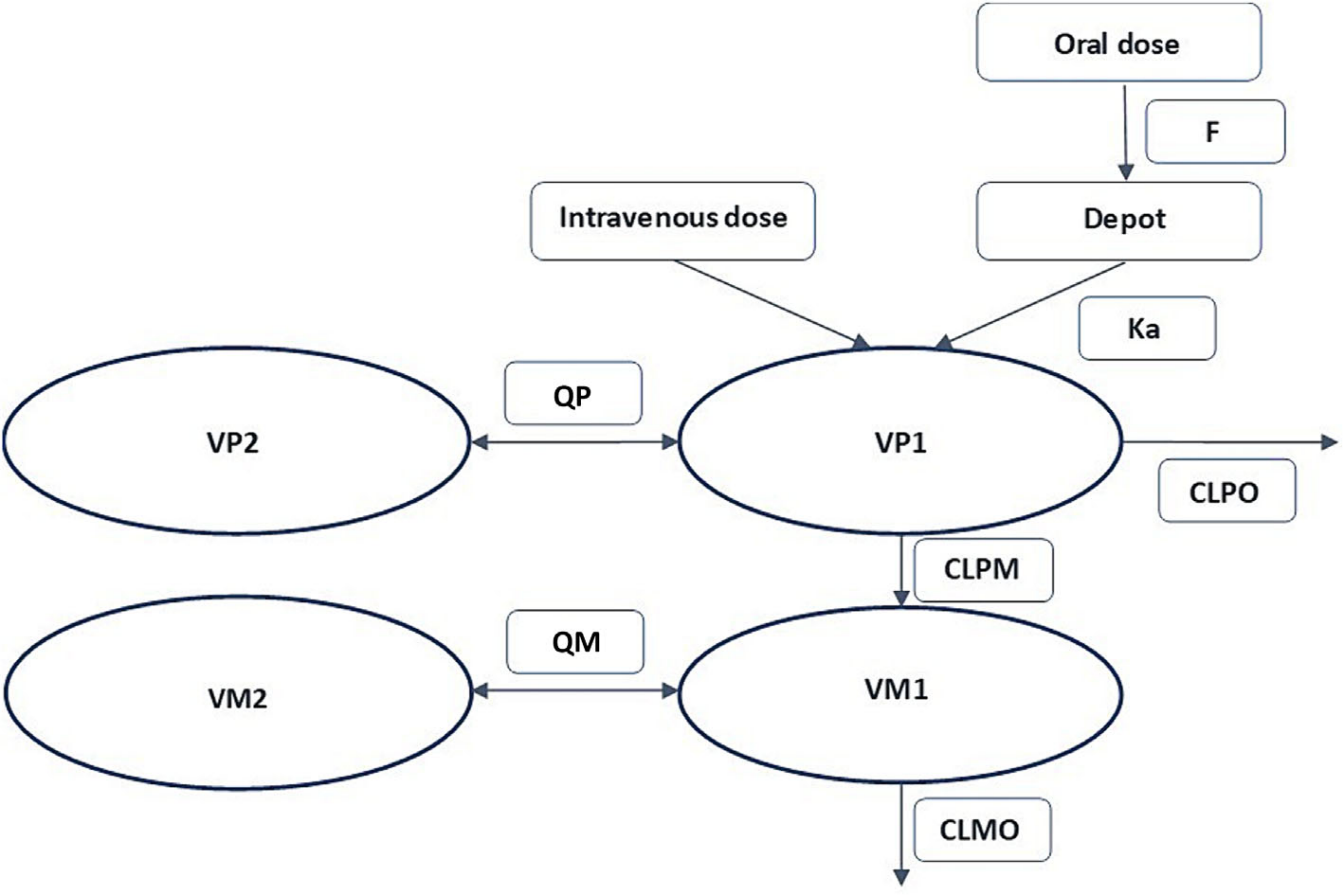
Pharmacokinetic model for tramadol and its active metabolite, O‐desmethyltramadol (M1). The two‐compartment linear‐disposition model describes the parent drug with inter‐compartmental clearance. Two additional compartments for the metabolite M1 are linked to the tramadol central compartment by M1 formation clearance. In the model, the total clearance of tramadol (CLPP) was first estimated independently, without considering M1. These values were then fixed in the subsequent steps to allow the estimation of the clearances of M1 (CLPM and CLMO). F, bioavailability; K_a_, absorption rate constant; CLPO, clearance of tramadol by other routes; QP, intercompartmental clearance; VP1, central volume of tramadol; VP2, peripheral volume of tramadol; CLMO, clearance of M1; CLPM, formation clearance to M1; VM1, central volume of M1; VM2, peripheral volume of M1; QM, intercompartmental clearance of M1.

Given the objectives of this investigation, an existing population pharmacokinetic model and its parameter estimates^20^ obtained from a wider population including children and adults were selected as priors for the evaluation of the effect of polymorphisms on the disposition of tramadol and M1. The model consists of two compartments for tramadol and two compartments for M1, having zero‐order input and first‐order elimination (Figure 2). Briefly, the model was parameterized in terms of clearance and volume of distribution standardized to a body weight of 70 kg according to an allometric function (Equation 1). In addition, a sigmoid maturation function described the age‐related changes in the clearance of tramadol (CLPP), the formation and elimination clearance of the metabolite (CLPM and CLMO, respectively) (Equation 2).

(1) $$F_{\text{size}}={\left(W_{i}/W_{\mathrm{STD}}\right)}^{\mathrm{PWR}}$$

where *W*_*i*_ is the weight of the *i* ^th^ individual. Allometric scaling was based on a power (*PWR*) exponent of 0.75 for clearance and 1 for volume of distribution.^20^ *F*_*size*_ is the allometrically scaled fraction of the parameter value in a subject with a standard weight of 70 kg, *W*_*STD*_.

(2) $$F_{\mathrm{mat}}=\frac{1}{1-{\left(\frac{\mathrm{PMA}}{{\mathrm{TM}}_{50}}\right)}^{\left(-\text{Hill}\right)}}$$

where *F*_*mat*_ is the maturation fraction relative to a mature adult; *PMA* is the postmenstrual age in weeks, *TM*_50_ is the *PMA* when clearance is at 50% of full maturity; and *Hill* is the exponent which modifies the steepness of the maturation curve.

Our main assumption for the approach presented here is that the effect of interindividual differences in disposition due to variation in CYP2D6 metabolic capacity and OCT1 transporter activity are described by the stochastic components and unexplained residual variability estimates from the initial model. Moreover, it was assumed that the magnitude of the effect associated with polymorphism was constant across age groups. Age (i.e., ontogeny) and weight‐related changes in pharmacokinetics were treated as independent factors.

The characterization of the effect of genetic covariates on drug disposition parameters was based on the phenotypes associated with activity scores (AS) for CYP2D6 and OCT1, i.e., poor metabolizers (AS 0), intermediate metabolizers (AS 0.5), extensive metabolizers (AS 1.0–2.0), and ultra‐rapid metabolizers (AS >2.0). Six genotype variants were identified in the original data, which we clustered into three groups, namely G**1** (AS 0 and 0.5), G**2** (AS 1 and 1.5) and G**3** (AS 2 and 3). This grouping was due to low patient numbers in some genotypes and supported by guidelines for CYP2D6 classification in codeine therapy.^25^ Following stratification of the data based on AS, the clearance of tramadol (CLPP), and the formation and elimination clearances of M1 (CLPM and CLMO) were estimated for each phenotype.

Initially, the PK parameters for tramadol were estimated independently from M1 data, with the addition of phenotype as a covariate on clearance parameters in a stepwise manner, including one gene at a time (e.g. CYP2D6). Tramadol‐specific parameters were then fixed in the subsequent step, during which M1 data were analysed concurrently with the parent drug. As data on the pharmacokinetics of M1 administration to humans was not available, estimates for the central volume of distribution (VM1) were fixed to a value of 78.9 L/70 kg. This value was scaled from a study in dogs, which evaluated the pharmacokinetics of M1 following intravenous administration.^27^ Prior estimates for intercompartmental clearance (QM) and peripheral volume of distribution were used from a prior study.^20^

To preserve stoichiometric correlations and the mass balance between parent drug and metabolite, differences in the molecular weight of the moieties must be taken into account. Consequently, to calculate accurate concentrations for both, a molar ratio of 0.947 was used during the integrated analysis. In addition, to ensure the model could be used to describe the PK profiles of tramadol following administration of oral drops and immediate release tablets, an absorption rate constant (K_a_) was added, with a population value of 1.28 h^−1^ obtained from a bioavailability study of tramadol capsules.^28^

### 2.4 Model evaluation and predictive performance

As indicated above, prior distributions were used as basis for the estimation of the fixed effects describing drug disposition characteristics.^29^, ^30^ Interindividual variability in PK parameters was assumed to be log‐normally distributed. A parameter value of an individual *i* (post‐hoc value) is therefore given by the following equation:

(3) $$\theta_{i}=\theta_{\mathrm{TV}}\times e^{\mathrm{\eta i}}$$

where *θ*_*TV*_ is the typical value of the parameter in the population and *ηi* is assumed to be a random variable with mean and variance (ω^2^) equal to zero. Residual variability, consisting of measurement and model error was described with a proportional and an additive error model. Therefore, for the *j* ^th^ observed concentration of the *i* ^th^ individual, residual error can be described by the following equations:

(4) $$Y_{\mathrm{ij}}=F_{\mathrm{ij}}+{Ꜫ}_{\mathrm{ij}}\times W$$

(5) $$Y=F+{Ꜫ}_{\mathrm{ADD}}$$

where *F*_*ij*_ is the predicted concentration with *ε*_*ij*_ as the random variable with mean zero and variance σ^2^. *W* is a proportional weighting factor for *ε*, and *Ꜫ*_*ADD*_ is the additive error (ng/mL).

Standard goodness‐of‐fit plots and statistical criteria were used as diagnostics for model selection. A decrease in objective function (OFV) of 3.89 points or more was considered as a statistically significant difference (*P* < .05 based on the χ^2^ distribution). Goodness‐of‐fit plots, including observed *vs*. individual predicted concentrations, observed *vs*. population predicted concentrations, conditional weighted residuals (CWRES) *vs*. time and conditional weighted residuals (CWRES) *vs*. population predicted concentrations were complemented by standardized visual predictive checks (VPC).

### 2.5 Simulation scenarios

Clinical trial simulations (CTS) were implemented using the final model parameter estimates obtained after incorporation of the effect of polymorphism on the formation and metabolic clearances of tramadol and M1. As outlined in Figure 1, oral tramadol doses ranging between 1 and 8 mg/kg/day were tested in a virtual cohort of patients aged between 3 months and 18 years old. Scenarios were based on a study protocol aimed at the treatment of chronic pain including a titration and a maintenance phase. Area under the concentration *vs*. time curves [AUC (ng*h/mL)], steady‐state concentrations [*C*_ss_ (ng/mL)] and peak concentrations [*C*_max_ (ng/mL)] were used as metrics of exposure. Simulation scenarios were evaluated to identify suitable doses and dosing regimens that minimize the proportion of patients whose exposure exceeds the predefined safety thresholds.

Based on preliminary work and taking into account the prevalence of nine subgroups (i.e., variants of CYP2D6 and OCT1 polymorphism combinations) in the population, cohorts of 100 patients for each simulation scenario were considered sufficient to assess the effect of the different covariates on systemic exposure to tramadol and M1. Demographic variables including age, sex and weight were obtained from the National Health and Nutrition Examination Survey (NHANES)^31^ and CALIPER^32^ databases. Altogether a large virtual population (*n* > 1000) was obtained, which we sampled from and stratified into 5 kg weight bands (*n* = 100), as summarized in Table 3.

**TABLE 3:** Demographic baseline characteristics of the virtual cohort used for the implementation of each simulation scenario.

Baseline characteristics	
Number of patients	100
Median PMA in weeks (range)	768 (51–976)
Median weight in kilograms (range)	45 (4.7–73.4)

### 2.6 Implications of genetic and non‐genetic, maturational factors on the exposure to tramadol and M1

A range of doses of tramadol was evaluated to assess the implications of different polymorphisms and other non‐genetic factors on the overall exposure to parent drug and M1 metabolite. A three times daily dosing regimen was used in conjunction with a titration schedule over a period of 3 weeks starting with 1 mg/kg/day, as shown in Table 4. Daily doses were also capped at 400 mg in accordance with current prescribing guidelines. The suitability of these doses, dosing regimen and interval between titration steps was evaluated taking into account the requirements for the assessment of the efficacy of gabapentin and tramadol in a prospective randomized study, under the auspices of the GAPP consortium.^33^

**TABLE 4:** Doses proposed for the titration phase of the GABA‐1 study protocol.^18^ The titration scheme was based on a starting dose of 1 mg/kg/day not exceeding the maximum limit of 400 mg/day. Up‐ and down‐titration rules considered both evidence of adequate analgesia and potential for adverse events. The pharmacokinetics of tramadol was to be assessed and analysed together with efficacy data at the end of the study.

Day 1	Day 3	Day 5	Day 14	Day 21
1 mg/kg/day	2 mg/kg/day	3 mg/kg/day	5 mg/kg/day	8 mg/kg/day

Given the clinical concern due to the increase in exposure to the active moieties in individuals with phenotype combinations CYP2D6 G3/OCT1 G0 and CYP2D6 G2/OCT1 G0, we have attempted to highlight the specific effect of these polymorphisms, by simulating all genotype combinations (CYP2D6 and OCT1) in a uniform cohort of patients (*n* = 100) with comparable demographic baseline characteristics. Predicted steady‐state concentrations were then used to calculate the proportion of patients exceeding the therapeutic safety threshold for tramadol and M1 by 10% or more (i.e., 330 ng/mL and 55 ng/mL, respectively). This rather conservative approach ensures that random fluctuations in plasma levels, in particular around peak concentrations, are taken into account. Results were stratified considering the metabolic phenotype of the variants that are likely to have clinical implications. Finally, to disentangle the effect of genotype/phenotype from that of maturation and developmental growth, steady state concentrations following a 1, 3 and 5 mg/kg are summarized by weight band (<10 kg, 10–< 20 kg, 20–<40 kg, >40 kg).

The evaluation of covariate effects was performed using a non‐linear mixed effects modelling approach with NONMEM version 7.3 (ICON Development Solutions, Ellicott City, MD, USA). The first‐order conditional estimation with interaction (FOCE‐I) option and ADVAN 6 TOL = 3 was used for model parameter estimation. All data processing steps, graphical and statistical summaries were implemented in R (version 3.6.1).^34^

### 2.7 Nomenclature of targets and ligands

Key protein targets and ligands in this article are hyperlinked to corresponding entries in the common portal for data from the IUPHAR/BPS Guide to PHARMACOLOGY,^35^ and are permanently archived in the Concise Guide to PHARMACOLOGY 2023/24.^36^

## 3 RESULTS

**FIGURE 3:**
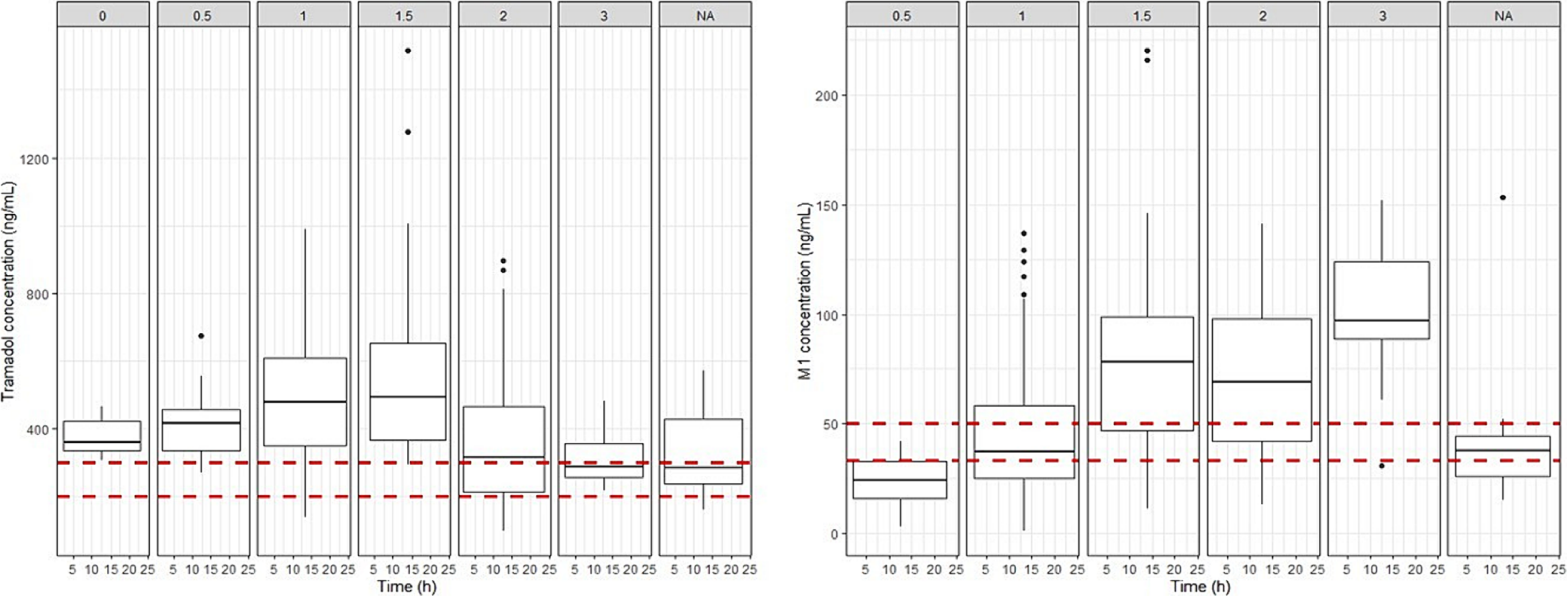
Steady‐state concentrations of tramadol (left) and M1 (right) following an average intravenous loading dose of 2.1 mg/kg over 30 min, followed by continuous infusion of 0.35 mg/kg/h (*n* = 52), stratified by activity scores for CYP2D6. NA (not available) panel denotes plasma concentrations where no CYP2D6 information was available. Whisker‐box plots show the median, quartiles and 95% prediction intervals. The red dotted lines display tramadol and M1's putative concentration ranges for analgesia (i.e., 200–300 ng/mL and 30–50 ng/mL, respectively).

Following an initial exploratory analysis (see Figure S1), it became evident that tramadol and M1 concentrations across all age groups were higher than what is generally considered efficacious for analgesic purposes (Figure 3). These patterns are further influenced by interindividual differences associated with polymorphisms in CYP2D6 and OCT1.

Despite high interindividual variability among patients, the pharmacokinetics of tramadol and M1 was adequately described by a two‐compartment model, with phenotype as a significant covariate in addition to the known effects of age (maturation) and body weight on the disposition of tramadol and its M1 metabolite. Initially, the effect of polymorphism in CYP2D6 was assessed on CLPP and CLPM, whereas the implications of OCT1 variants was evaluated on CLMO. Following the stepwise covariate model building, the effect of polymorphism was found to be significant on CLPP, CLPM, and CLMO, with CYP2D6 influencing CLPP and CLPM, and OCT1 affecting CLMO. The diagnostic plots (Figures S2 and S3) show that the final model adequately explained the variability in the data, producing unbiased population and individual predictions (Figures S4 and S5). No significant correlations or trends were noted between the conditional weighted residuals or body weight. Visual predictive checks of tramadol and its metabolite (Figure S6) provide further evidence of the predictive performance and adequacy of the final model including the contribution of the different genotypes to the known effect of maturation (ontogeny) and body weight on the disposition properties of the two moieties. The final parameters for both moieties are shown in Table 5. Full details of the model parameterization, along with the control stream file can be found in the Supporting Information.

**TABLE 5:** Pharmacokinetic parameter estimates of tramadol and O‐desmethyltramadol used in the different simulation scenarios.

Estimate	Units	Point estimate	RSE (%)
CLPP (CYP2D6‐G1)	L/h/70 kg	28.7	11.7
CLPP (CYP2D6‐G2)	L/h/70 kg	36.3	7.8
CLPP (CYP2D6‐G3)	L/h/70 kg	43	7.9
CLPO	L/h/70 kg	25.6	10
QP	L/h/70 kg	111	58.3
VP1	L/70 kg	141	62.2
VP2	L/70 kg	102	73.6
CLMO (OCT1‐G0)	L/h/70 kg	57.7	35
CLMO (OCT1‐G1)	L/h/70 kg	78.7	34
CLMO (OCT1‐G2)	L/h/70 kg	62.2	32
QM	L/h/70 kg	635^a^	‐
VM1	L/70 kg	78.9^a^	‐
VM2	L/70 kg	101	101
CLPM (CYP2D6‐G1)	L/h/70 kg	5.08	35
CLPM (CYP2D6‐G2)	L/h/70 kg	11.4	35
CLPM (CYP2D6‐G3)	L/h/70 kg	14.7	32
TM50 CLPO	Weeks (PMA)	39.1^a^	‐
Hill coefficient CLPO	‐	6.76^a^	‐
TM50 CLPM	Weeks (PMA)	39.8^a^	‐
Hill coefficient CLPM	‐	9^a^	‐
TM50 CLMO	Weeks (PMA)	47.7^a^	‐
Hill coefficient CLMO	‐	3.4^a^	
**Between‐subject variability (CV%)**			
VP1	‐	0.211	
CLPO (CYP1)	‐	0.161	28
CLPM (CYP1)	‐	0.628	42
CLPM (CYP2)	‐	0.0915	47
CLPM (CYP3)	‐	0.00878	25
QM	‐	1.98^a^	‐
VM1	‐	1.45^a^	‐
**Residual variability**			
Proportional error (tramadol)		0.0159	12
Additive error (tramadol)	ng/mL	0.01	45
Proportional error (M1)		0.00001	11
Additive error (M1)	ng/mL	0.00004	42

a Values were fixed using the estimates from Allegaert et al.^19^

Abbreviations: CLMO, clearance of M1; CLPM, formation clearance to M1; CLPO, clearance of tramadol by other routes; CLPP, total clearance of tramadol; QM, intercompartmental clearance of M1; QP, intercompartmental clearance of tramadol; RSE, relative standard error; VM1, central compartment volume of M1; VM2, peripheral compartment volume of M1; VP1, central compartment volume of tramadol; VP2, peripheral compartment volume of tramadol.

### 3.1 Simulation scenarios

**FIGURE 4:**
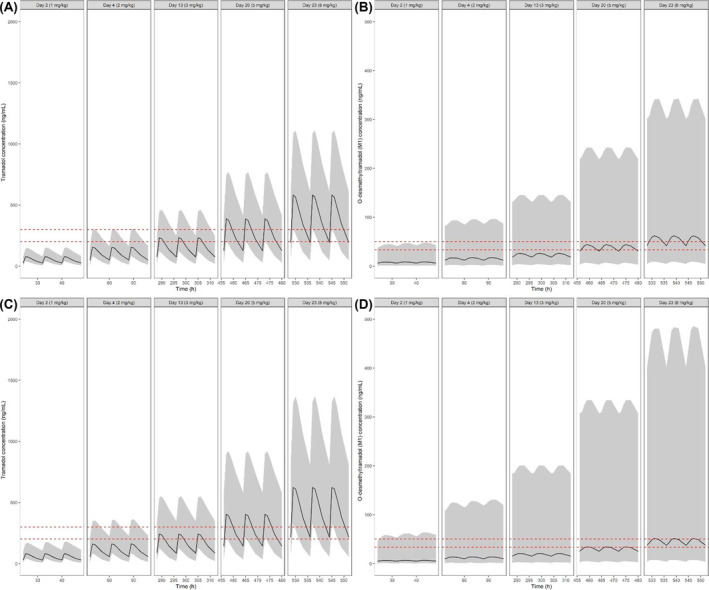
Predicted tramadol and M1 concentration *vs.* time profiles after each titration step for polymorphism interactions of clinical concern, i.e., individuals whose phenotype is associated with extensive or ultra‐rapid CYP2D6 metabolism and low OCT1 transporter activity. Panels A and B depict CYP2D6 Group 3 and OCT1 Group 0. Panels C and D depict CYP2D6 Group 2 and OCT1 Group 0. The red dotted lines display tramadol and M1's putative efficacious concentration ranges for analgesia (*n* = 900; PMA = 51–976 weeks; weight = 4.7–73.4 kg). Black solid lines are median concentrations. Shaded areas show the 95% prediction intervals. Summary statistics of these profiles, including area under the concentration *vs.* time curves [AUC (ng*h/mL)], steady‐state concentrations [*C*_ss_ (ng/mL)] and peak concentrations [*C*_max_ (ng/mL)] for each titration step are shown in Table S1.

An attempt was made to characterize the effect of the combination of specific phenotypes, which could have clinical safety implications in case of inadequate use or overdosing. Figure 4 shows the predicted time course of tramadol and M1 concentration for CYP2D6 Group 3 and OCT1 Group 0 (panels A and B) and CYP2D6 Group 2 and OCT1 Group 0 (panels C and D) following the different titration steps, as proposed in the GABA‐1 study protocol.^33^ The occurrence of these two phenotype combinations appears to be the ones of clinical concern. Summary statistics for AUC, *C*_ss_ and *C*_max_ after each titration step are presented in Table S1.

Clearly, the increase in systemic exposure to tramadol and M1 associated with such a combination exceeds by far the effect of interindividual differences in drug disposition due to maturation or developmental growth in children. It can be noted that as early as the second titration step of 2 mg/kg, subjects with these phenotypical variants are likely to be exposed to levels of both moieties in excess of that required for analgesia. An overview of the other genotype/phenotype combinations are provided in the Supporting Information (Figures S7 and S8).

Based on the simulated scenarios, it was possible to assess the proportion of patients that exceeded the putative therapeutic threshold in each titration step. These results provide insight into the probability of observing adverse events due to potential overexposure to tramadol and/or its M1 metabolite. As summarized in Tables 6 and 7, steady state concentrations of tramadol above the reference safety threshold are observed in ≥5% of subjects for doses of 3 mg/kg or higher. Similarly, for O‐desmethyltramadol, exposure above the proposed threshold of 55 ng/mL is observed in ≥5% of the subjects receiving tramadol doses of 2 mg/kg or higher.

**TABLE 6:** Predicted number and percentage (%) of subjects in the simulated cohort (*n* = 900) exceeding the proposed safety threshold across the different CYP2D6/OCT1 polymorphism combinations stratified by dose level. Results are based on the assumption of steady‐state conditions. These results should be interpreted carefully, as they also include the effect of age and body weight on the disposition of tramadol and its metabolite.

Dose of tramadol (mg/kg/day)	No. (%) of patients > 330 ng/mL tramadol	No. (%) of patients > 55 ng/mL M1
1	1 (0.1)	27 (3.0)
2	36 (4.0)	89 (9.9)
3	136 (15.1)	143 (15.9)
5	422 (46.9)	242 (26.9)
8	637 (70.7)	333 (37.0)

**TABLE 7:** A breakdown of the combination of different polymorphisms in the simulated cohort of patients (*n* = 900). Data summarize the percentage of subjects who exceed the proposed safety threshold of 330 ng/mL for tramadol and 55 ng/mL for O‐desmethyltramadol (M1). These results should be interpreted carefully, as they also include the effect of age and body weight on the disposition of tramadol and its metabolite.

Dose (mg/kg)	CYP2D6 variant^a^	OCT1 variant^a^	Percentage of subjects above the reference safety threshold (%)	
			Tramadol	O‐desmethyltramadol
1	CYP2D6‐G1	OCT1‐G0	1	3
		OCT1‐G1	0	1
		OCT1‐G2	0	2
1	CYP2D6‐G2	OCT1‐G0	0	3
		OCT1‐G1	0	1
		OCT1‐G2	0	3
1	CYP2D6‐G3	OCT1‐G0	0	4
		OCT1‐G1	0	3
		OCT1‐G2	0	7
2	CYP2D6‐G1	OCT1‐G0	8	5
		OCT1‐G1	4	8
		OCT1‐G2	0	6
2	CYP2D6‐G2	OCT1‐G0	4	14
		OCT1‐G1	2	5
		OCT1‐G2	2	7
2	CYP2D6‐G3	OCT1‐G0	6	14
		OCT1‐G1	1	11
		OCT1‐G2	4	19
3	CYP2D6‐G1	OCT1‐G0	5	8
		OCT1‐G1	23	13
		OCT1‐G2	19	11
3	CYP2D6‐G2	OCT1‐G0	19	20
		OCT1‐G1	15	10
		OCT1‐G2	14	16
3	CYP2D6‐G3	OCT1‐G0	16	18
		OCT1‐G1	10	20
		OCT1‐G2	9	27
5	CYP2D6‐G1	OCT1‐G0	11	16
		OCT1‐G1	58	18
		OCT1‐G2	60	16
5	CYP2D6‐G2	OCT1‐G0	49	28
		OCT1‐G1	37	28
		OCT1‐G2	49	23
5	CYP2D6‐G3	OCT1‐G0	53	33
		OCT1‐G1	35	36
		OCT1‐G2	39	44
8	CYP2D6‐G1	OCT1‐G0	42	22
		OCT1‐G1	84	25
		OCT1‐G2	80	21
8	CYP2D6‐G2	OCT1‐G0	72	44
		OCT1‐G1	62	36
		OCT1‐G2	73	38
8	CYP2D6‐G3	OCT1‐G0	75	49
		OCT1‐G1	62	45
		OCT1‐G2	65	53

^a^ The use of groups to describe the different variants as discrete covariates on pharmacokinetic parameters was required due to the low prevalence of certain polymorphic variants in the population. CYP2D6 groups G1, G2 and G3, as well as OCT1 groups G0, G1 and G2 are based on activity scores (AS), as shown in Table 2.

Finally, for the two subgroups of clinical concern, stratification of the secondary PK parameters by weight bands (<10 kg, 10–<20 kg, 20–<40 kg, >40 kg) allow us to disentangle the effect of genotype/phenotype from that of maturation and developmental growth (Table S2).

## 4 DISCUSSION

Therapeutic choices for optimal analgesia in chronic pain in children are limited by the lack of suitable medicinal products with an approved label for this population. Furthermore, expansion of the label indication for medicinal products approved before the introduction of the paediatric legislation, i.e., off‐patent drugs, has been very limited due to lack of incentives.^37^ This has been further compounded by practical and operational challenges in the implementation of randomized controlled studies in children,^38^ as alternative methods for supporting evidence of efficacy and safety have not been deemed sufficient by regulators. Indeed, even a drug approved in the mid‐1990s such as tramadol does not yet have a unanimous dosing recommendation or consensus regarding its use for the treatment of paediatric patients across European countries or in the United States. Yet, since 2012, it is preferred to codeine following recommendations against the latter's use in paediatric analgesia protocols.^39^ Even though it has been placed into schedule IV of the Controlled Substances Act (CSA) in 2014, tramadol remains the second most prescribed analgesic product in the US. In the UK, tramadol use is still relatively widespread despite prescribing having become more restricted since 2012.^40^ Unfortunately, attempts to identify opportunities to optimize and/or personalize the dose for an improved benefit–risk profile have been scarce and limited to descriptive summaries of safety and efficacy in acute, postoperative pain.^41^, ^42^, ^43^

Here, we have shown how a model‐based approach can be used to characterize the effect of genetic and non‐genetic factors on drug disposition and to assess the implications of polymorphism in drug metabolism and transport mechanisms. Most importantly, this approach has allowed us to disentangle the contribution of baseline patient characteristics (i.e., intrinsic factors) from drug‐specific factors (e.g. formulation), known to determine interindividual variability in exposure and overall analgesic response to tramadol. Of note is the realization that in neonates, phenotypical differences further contribute to the effect of ontogeny of metabolizing enzymes (Figure 4). This effect is augmented by developmental growth with increasing body weight, further affecting drug disposition in older children. Consequently, insights from this analysis can guide the rationale for an optimized dosing regimen for tramadol in the paediatric population. In addition, it also enables the design of a prospective protocol, including genotyping requirements along with an appropriate risk mitigation strategy.

Our analysis reveals that despite previous data showing that maturation of the metabolic clearance of tramadol is complete by approximately 44 weeks post‐conception,^20^ current prescribing guidelines do not take into account the effects of its M1 metabolite, which has greater potency and contributes not only to interindividual variability in drug exposure, but also in the analgesic effect. This is an important shortcoming, given that the variation in organ maturation (which determines M1 formation) represents a risk factor for overdosing of young infants.

Regardless of the lack of controlled efficacy studies with tramadol in chronic pain in the paediatric population,^6^ there is no evidence suggesting significant difference in the pharmacological mechanisms and underlying pharmacokinetic‐pharmacodynamic relationships for analgesia in adults and children. Therefore, in the absence of pharmacodynamic data or clinical response (e.g., pain scale or scoring), it is plausible to assume that pharmacokinetics can be used as a proxy for pain relief and analgesia, and as such support not only the selection of a dose, but also personalization of dosing regimens. This working hypothesis contributes to reducing the empiricism of overt symptom relief as the primary measure of efficacy, upon which paediatric clinical trials are often based.

Clearly, one may question whether genotyping is feasible and worth the additional burden when alternative medicines can be prescribed, or whether genotyping is more relevant to acute or chronic treatment. A personalized approach for chronic pain interventions may benefit from genotype testing before treatment initiation, as it would give practitioners a scientific basis for accurate prescribing. Over‐ and underdosing of patients due to lack of efficacy/tolerance should be minimized or prevented throughout chronic treatment with opioids. While recommendations for the use of tramadol in acute pain rely on treatment initiation at the lowest possible dose with adjustment according to efficacy and tolerability criteria, it is argued that it is worthwhile to determine the CYP2D6 genotype activity before commencing tramadol in the case of chronic treatment. One reason for the use of up‐titration and tapering procedures is that acute/inpatient treatments are closely monitored by clinicians as opposed to outpatient prescriptions. By contrast, the use of a target concentration intervention (TCI) in chronic pain could offer an alternative, more integrated strategy to genotyping and therapeutic drug monitoring (TDM).^44^

As hypothesized previously, it has been assumed that tramadol concentrations of 200–300 ng/mL can be considered as an analgesic target range.^26^ Interestingly, in a study by Lehman and colleagues,^45^ the minimum effective tramadol serum concentration varied greatly, with median concentrations of 287.7 ng/mL and 36.2 ng/mL for tramadol and M1, respectively. In another study,^46^ mean tramadol and M1 plasma concentrations of 590 and 84 ng/mL were associated with adequate analgesia when adults were responsible for self‐administration of the drug (patient‐controlled analgesia), which could suggest that analgesic levels of tramadol may not be accurately interpreted without taking into account the exposure to M1 metabolite. Analgesia appears, therefore, to depend on the exposure parent drug and circulating metabolite. This evidence highlights the role of genotyping prior to prescribing tramadol, as well as the need to consider TDM after initiating treatment in paediatric patients requiring chronic analgesia.

We also acknowledge a number of limitations in our analysis. For completeness, the key points are summarized in the Supporting Information. Despite the limitations, our simulation results suggest the need for slow, stepwise titration to effect and capping of the maximum dose of tramadol to be used in children with body weight <40 kg at 5 mg/kg up to a maximum of 400 mg for those with higher body weight. As this investigation was triggered by an agreed paediatric investigation plan, in which the efficacy of gabapentin is to be compared with tramadol and morphine,^18^, ^33^, ^47^ we expect that evidence arising from this prospective study with a larger number of subjects will substantiate the current findings and establish the benefit–risk ratio for the paediatric population affected by chronic pain.

In summary, to date high‐quality evidence supporting the treatment of chronic pain in paediatric patients remains sparse and clinical practice relies primarily on expert opinion (i.e., evidence level 4).^48^ Opioids have been considered for treatments that last as short as possible, but no quantitative evaluation has been made of the underlying benefit–risk balance for their use in chronic conditions.^49^ In this context, our investigation represents a first step for the generation of good quality evidence, providing a robust scientific basis for the dose rationale for treatment of chronic pain in children and young people. It was aimed to support the dose rationale in a prospective, randomized clinical study, but our findings are likely to be relevant for prescribers and paediatricians considering oral doses of tramadol in a real‐life setting. Uptitration of tramadol is recommended to minimize the risks associated with potentially high exposure to O‐desmethyltramadol in patients with certain genetic phenotypes. A starting dose of 0.5 mg/kg followed by stepwise increases of 1 mg/kg up to a maximum of 5 mg/kg (capped to 400 mg) should warrant an adequate safety profile, even when individual genotype information is not readily available.

## AUTHOR CONTRIBUTIONS

Paul Healy performed the data analysis, wrote and reviewed the manuscript; Karel Allegaert contributed to the interpretation of the results and reviewed the manuscript; Oscar Della Pasqua designed the study, contributed to the interpretation of the results and reviewed the manuscript.

## CONFLICT OF INTEREST STATEMENT

All authors declare no competing interests for this work.

## Supporting information


**Figure S1.** Observed tramadol (left panel) and M1 (right panel) concentrations in neonatal patients (*n* = 52) at different sampling times and intravenous doses of tramadol (average loading dose of 2.1 mg/kg over 30 min, followed by continuous infusion of 0.35 mg/kg/h), stratified by activity scores for CYP2D6. NA (not available) panel denotes plasma concentrations where no CYP2D6 information was available. Dashed red lines indicate the putative therapeutic range for tramadol (200–300 ng/mL) and M1 (30–50 ng/mL).
**Figure S2.** Diagnostic plots for the final pharmacokinetic model for tramadol in neonates. (A) Observed *vs.* population predicted concentrations, (B) observed *vs.* individual predicted concentrations, (C) conditional weighted residuals *vs.* population predicted concentrations and (D) conditional weighted residuals *vs.* time. The black line represents the line of unity and the red line is the loess curve.
**Figure S3.** Diagnostic plots for the final pharmacokinetic model for O‐desmethyltramadol in neonates. (A) Observed *vs.* population predicted concentrations, (B) observed *vs.* individual predicted concentrations, (C) conditional weighted residuals *vs.* population predicted concentrations and (D) conditional weighted residuals *vs.* time. The black line represents the line of unity and the red line is the loess curve.
**Figure S4.** Individual (solid blue line) and population (solid red line) predicted tramadol concentration *vs.* time profiles. Dots represent the observed concentrations of tramadol in neonatal and infant patients.
**Figure S5.** Individual (solid blue line) and population (solid red line) predicted O‐desmethyl‐tramadol concentration *vs.* time profiles. Dots represent the observed concentrations of tramadol in neonatal and infant patients.
**Figure S6.** Visual predictive check (VPC) plots for tramadol (left panel) and O‐desmethyltramadol (right panel). Dots indicate the observed concentrations in the study population (*n* = 46). Solid lines depict the median, 5^th^ and 95^th^ percentiles of the observed plasma concentrations. The shaded area describes the 90% confidence interval around the model‐predicted median, 5^th^ and 95^th^ percentiles based on 500 simulations.
**Figure S7.** Predicted tramadol concentration *vs.* time profiles for different genotype/phenotype groupings following each titration step. In each panel, the solid lines represent the median of the simulated profiles along with the corresponding 95% prediction intervals depicted in the shaded areas. The red dotted lines represent tramadol's putative concentration range for analgesia (*n* = 900; PMA = 51–976; weight = 4.7–73.4). See Table 2 in the main manuscript for the criteria used for the different groups used as discrete covariates for modelling and simulation purposes.
**Figure S8.** Predicted O‐desmethyltramadol concentration *vs.* time profiles for different genotye/phentotype groupings following each titration step with oral tramadol. In each panel, the solid lines represent the median of the simulated profiles along with the corresponding 95% prediction intervals depicted in the shaded areas. The red dotted lines display M1's putative efficacious concentration window for analgesia (*n* = 900; PMA = 51–976; weight = 4.7–73.4). See Table 2 in the main manuscript for the criteria used for the different groups used as discrete covariates for modelling and simulation purposes.
**Table S1.** Summary statistics for tramadol (upper panel) and O‐desmethyltramadol (M1) (lower panel). AUC, *C*_ss_ and *C*_max_ are shown for patients with polymorphism combinations of clinical concern (CYP2D6‐ G3/OCT1‐G0 and CYP2D6‐G2/OCT1‐G0)* following stratification according to a titration schedule including oral doses of 1, 2, 3, 5 and 8 mg/kg tramadol. Values are medians and 90% prediction intervals. See Figure 4 for the time course of concentrations of tramadol and its metabolite M1 in these subgroups.
**Table S2.** Secondary pharmacokinetic parameters stratified by weight bands in paediatric patients with clinically relevant metabolic genotype/phenotype variants. Values are medians and 90% prediction interval.

## Data Availability

The data that support the findings of this study are available from the corresponding author upon reasonable request. Karel Allegaert is the custodian of the raw data.
