# Supplementary material for: Evaluation of the effect of CYP2D6 and OCT1 polymorphisms on the pharmacokinetics of tramadol: Implications for clinical safety and dose rationale in paediatric chronic pain
Source: Br J Clin Pharmacol. 2024 Oct 9;91(2):283–96. doi: 10.1111/bcp.16201 (PMC11773095; doi:10.1111/bcp.16201)
Supplement: Supplementary file 1 — Figure S1. Observed tramadol (left panel) and M1 (right panel) concentrations in neonatal patients (n = 52) at different sampling times and intravenous doses of tramadol (average loading dose of 2.1 mg/kg over 30 min, followed by continuous infusion of 0.35 mg/kg/h), stratified by activity scores for CYP2D6. NA (not available) panel denotes plasma concentrations where no CYP2D6 information was available. Dashed red lines indicate the putative therapeutic range for tramadol (200–300 ng/mL) and M1 (30–50 ng/mL). Figure S2. Diagnostic plots for the final pharmacokinetic model for tramadol in neonates. (A) Observed vs. population predicted concentrations, (B) observed vs. individual predicted concentrations, (C) conditional weighted residuals vs. population predicted concentrations and (D) conditional weighted residuals vs. time. The black line represents the line of unity and the red line is the loess curve. Figure S3. Diagnostic plots for the final pharmacokinetic model for O‐desmethyltramadol in neonates. (A) Observed vs. population predicted concentrations, (B) observed vs. individual predicted concentrations, (C) conditional weighted residuals vs. population predicted concentrations and (D) conditional weighted residuals vs. time. The black line represents the line of unity and the red line is the loess curve. Figure S4. Individual (solid blue line) and population (solid red line) predicted tramadol concentration vs. time profiles. Dots represent the observed concentrations of tramadol in neonatal and infant patients. Figure S5. Individual (solid blue line) and population (solid red line) predicted O‐desmethyl‐tramadol concentration vs. time profiles. Dots represent the observed concentrations of tramadol in neonatal and infant patients. Figure S6. Visual predictive check (VPC) plots for tramadol (left panel) and O‐desmethyltramadol (right panel). Dots indicate the observed concentrations in the study population (n = 46). Solid lines depict the median, 5th and 95th [file BCP-91-283-s001.pdf]

Supporting Information

Evaluation of the effect of CYP2D6 and OCT1 polymorphisms on the pharmacokinetics of tramadol: implications for clinical safety and dose rationale in paediatric chronic pain.

Paul Healy[1], Karel Allegaert[2,3,4] and Oscar Della Pasqua[1,5*]

[1] Clinical Pharmacology & Therapeutics Group University College ,London, London, UK.

[2] Department of Development and Regeneration, KU Leuven, Leuven, Belgium.

[3] Department of Pharmaceutical and Pharmacological Sciences, KU Leuven, Leuven, Belgium.

[4] Department of Hospital Pharmacy, Erasmus MC, Rotterdam, The Netherlands.

[5] Clinical Pharmacology Modelling and Simulation, GlaxoSmithKline, London, UK.

[*]Corresponding author:
Prof Oscar Della Pasqua
Clinical Pharmacology & Therapeutics Group
University College London
BMA House
Tavistock Square
London WC1H 9JP
United Kingdom
Email: o.dellapasqua@ucl.ac.uk
Phone: +4420778741544

ORCID identifiers:
Karel Allegaert – 0000-0001-9921-5105
Oscar Della Pasqua – 0000-0002-6211-1430

Keywords: tramadol, analgesia, chronic pain, paediatrics, personalised dose, extrapolation, CYP2D6, OCT1.

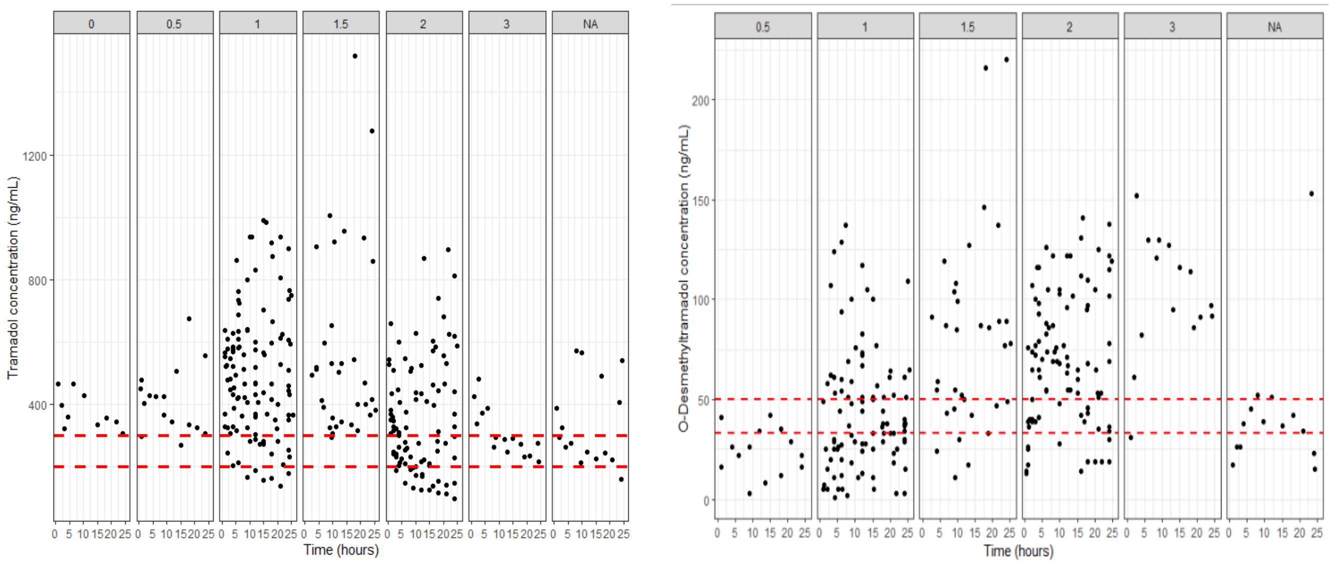

Figure S1. Observed tramadol (left panel) and M1 (right panel) concentrations in neonatal patients (N = 52) at different sampling times and intravenous doses of tramadol (average loading dose of 2.1 mg/kg over 30 minutes, followed by continuous infusion of 0.35 mg/kg/h), stratified by activity scores for CYP2D6 (NA, not available) panel denotes plasma concentrations where no CYP2D6 information was available). Dashed red lines indicate the putative therapeutic range for tramadol (200-300 ng/mL) and M1 (30-50 ng/mL).

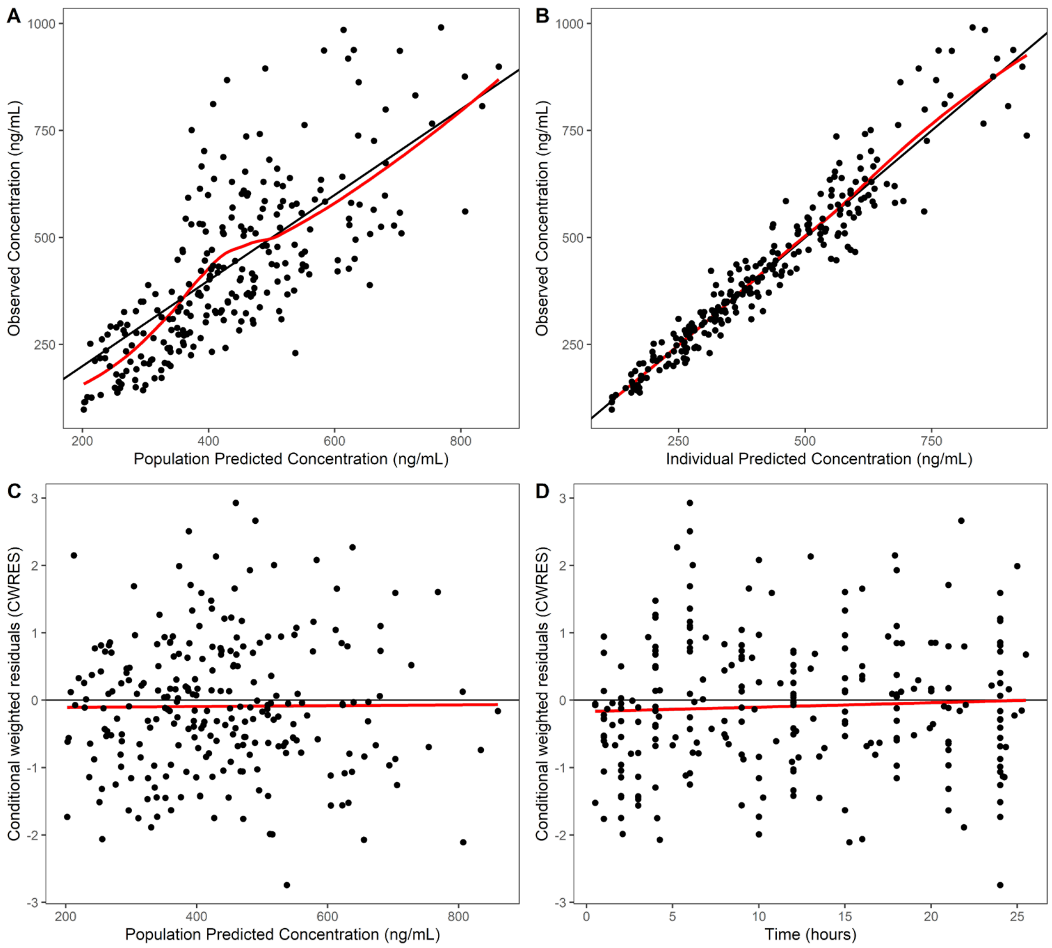

Figure S2. Diagnostic plots for the final pharmacokinetic model for tramadol in neonates. (A) Observed vs. population predicted concentrations, (B) observed vs. individual predicted concentrations, (C) conditional weighted residuals vs. population predicted concentrations and (D) conditional weighted residuals vs. time. The black line represents the line of unity and the red line is the loess curve.

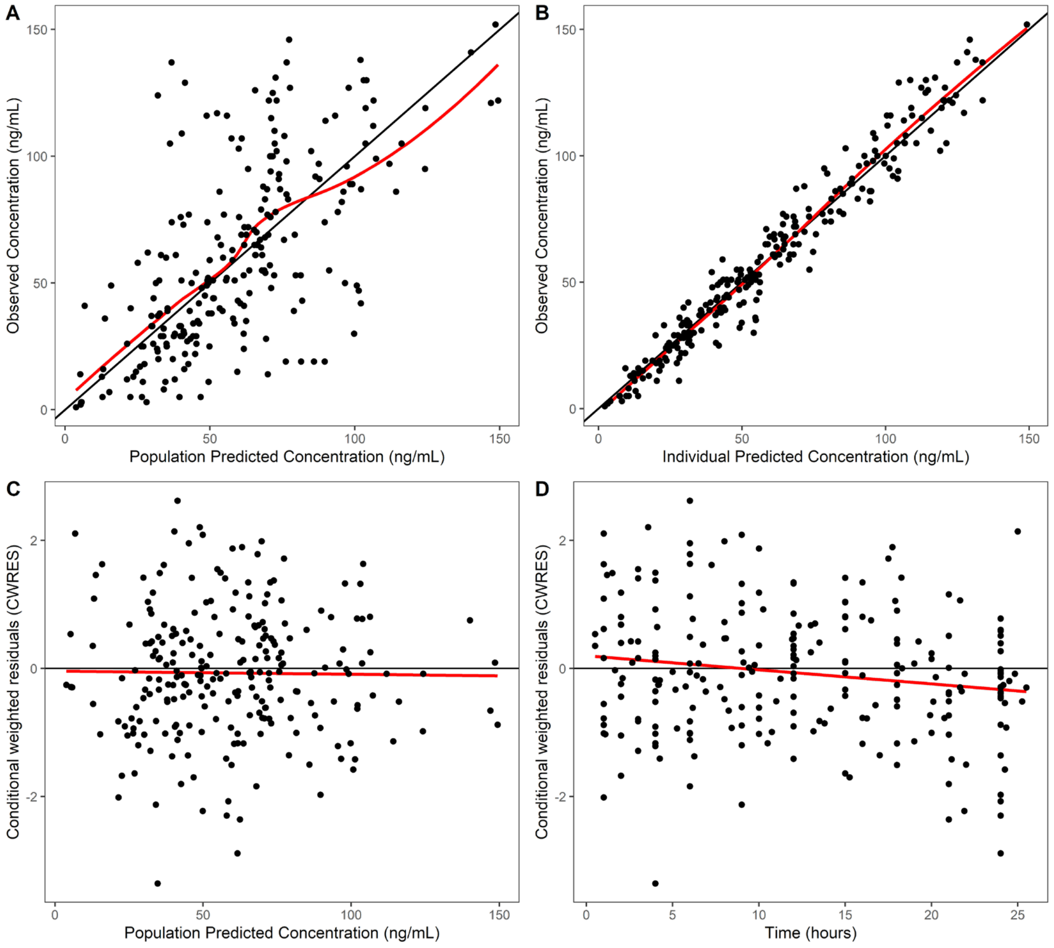

Figure S3. Diagnostic plots for the final pharmacokinetic model for O-desmethyltramadol in neonates. (A) Observed vs. population predicted concentrations, (B) observed vs. individual predicted concentrations, (C) conditional weighted residuals vs. population predicted concentrations and (D) conditional weighted residuals vs. time. The black line represents the line of unity and the red line is the loess curve.

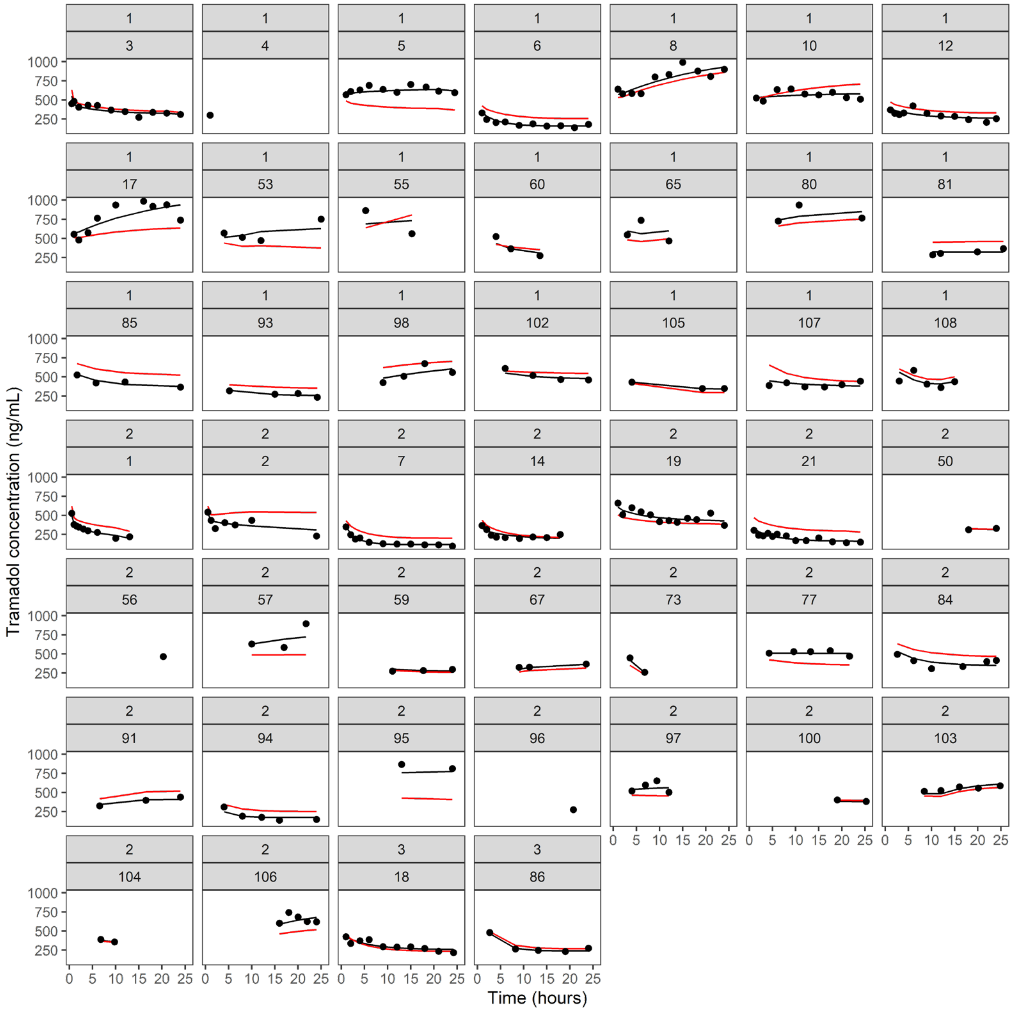

Figure S4. Individual (solid blue line) and population (solid red line) predicted tramadol concentration versus time profiles. Dots represent the observed concentrations of tramadol in neonatal and infant patients.

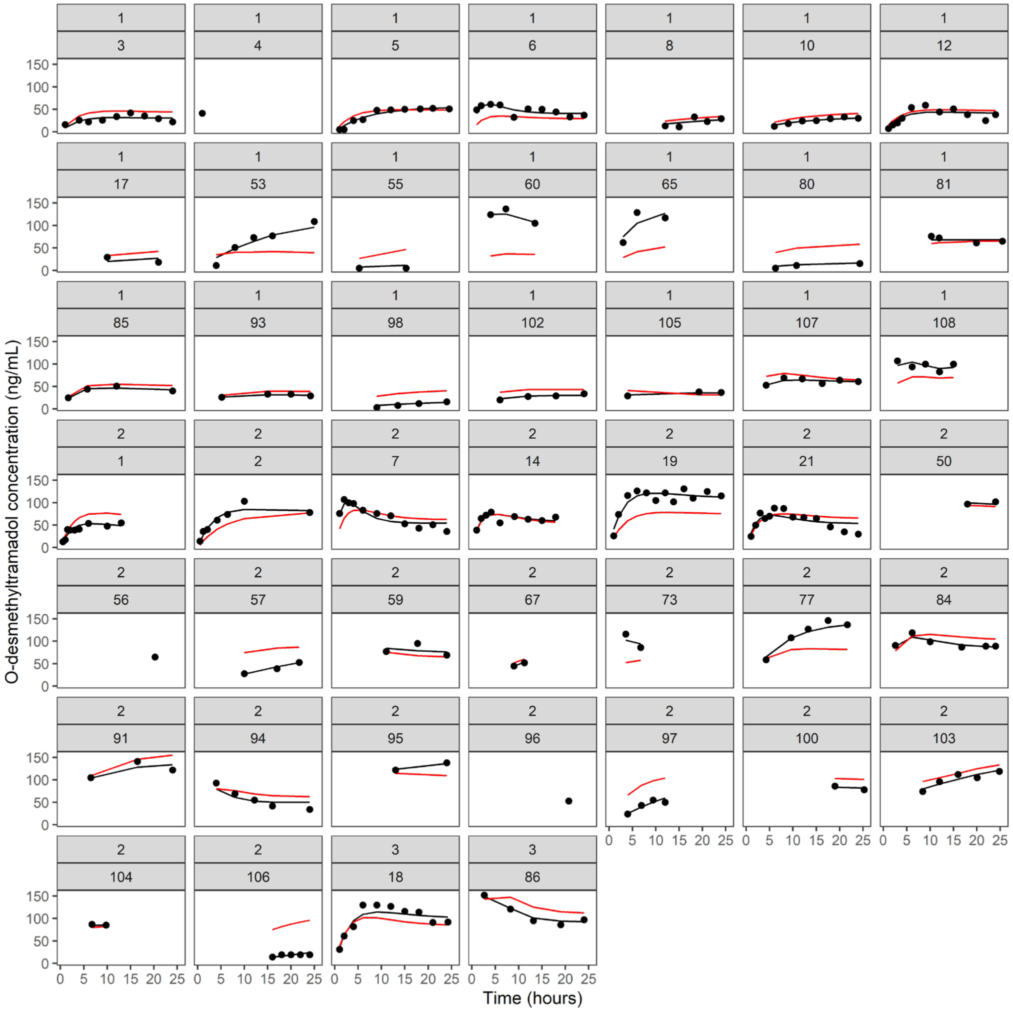

Figure S5. Individual (solid blue line) and population (solid red line) predicted O-desmethyl-tramadol concentration versus time profiles. Dots represent the observed concentrations of tramadol in neonatal and infant patients.

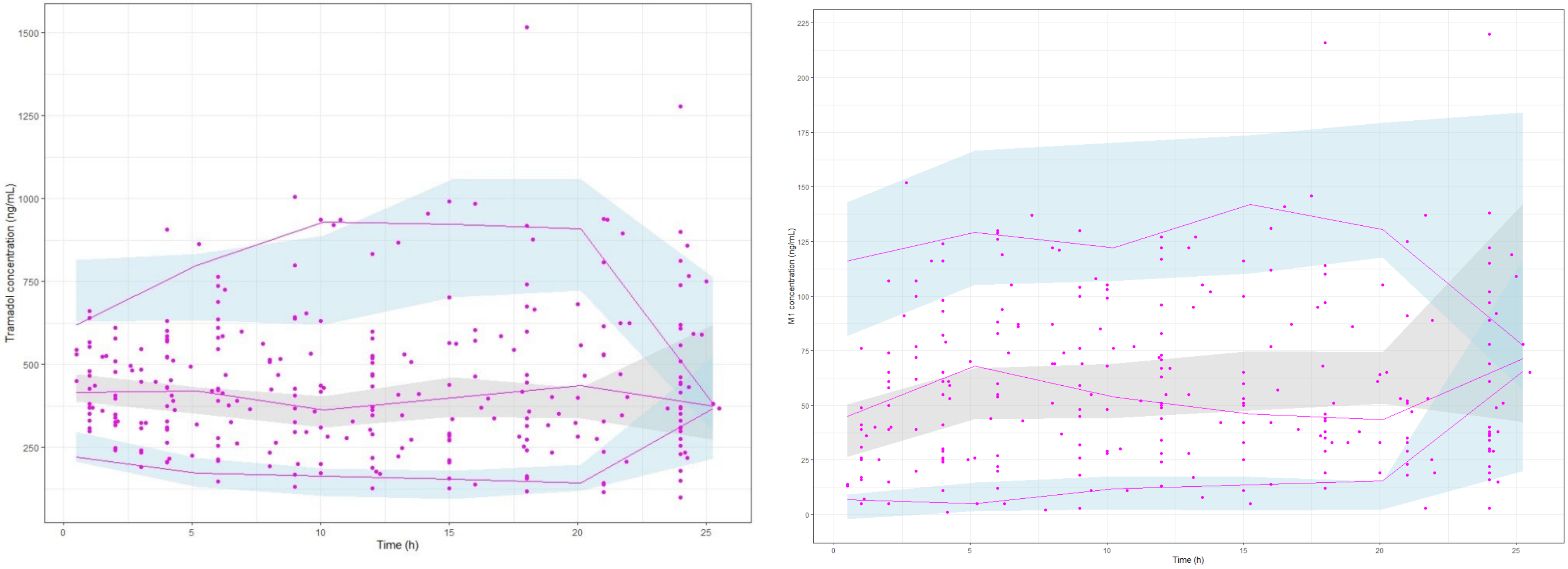

Figure S6. Visual predictive check (VPC) plots for tramadol (left panel) and O-desmethyltramadol (right panel). Dots indicate the observed concentrations in the study population (n=46). Solid lines depict the median, 5th and 95th percentiles of the observed plasma concentrations. The shaded area describes the 90%-confidence interval around the model-predicted median, 5th and 95th percentiles based on 500 simulations.

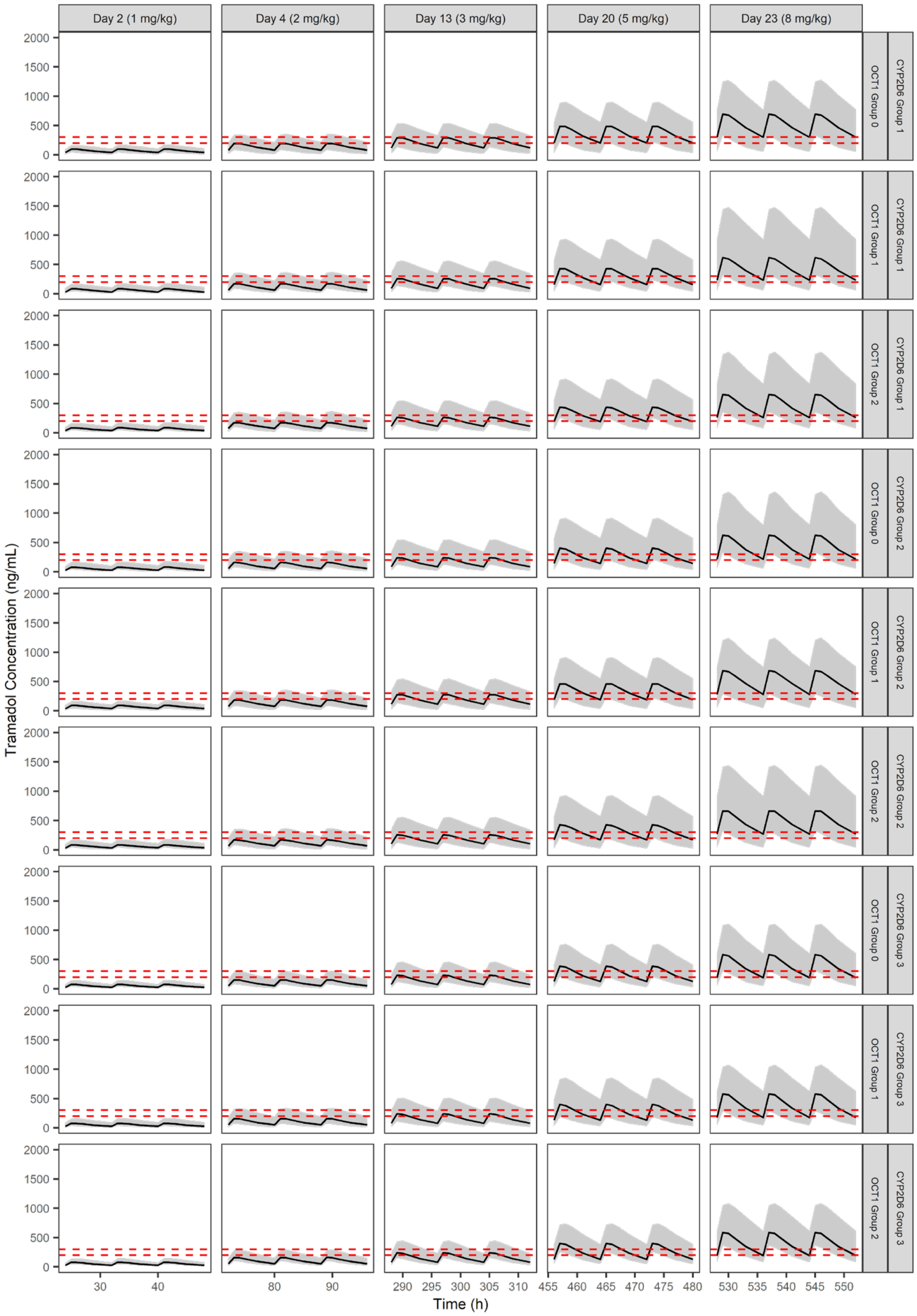

Figure S7. Predicted tramadol concentration vs time profiles for different genotype/phenotype groupings following each titration step. . In each panel, the solid lines represent the median of the simulated profiles along with the corresponding 95% prediction intervals depicted in the shaded areas. The red dotted lines represent tramadol's putative concentration range for analgesia (n=900; PMA = 51-976; weight = 4.7-73.4). See Table 2 in the main manuscript for the criteria used for the different groups used as discrete covariates for modelling and simulation purposes.

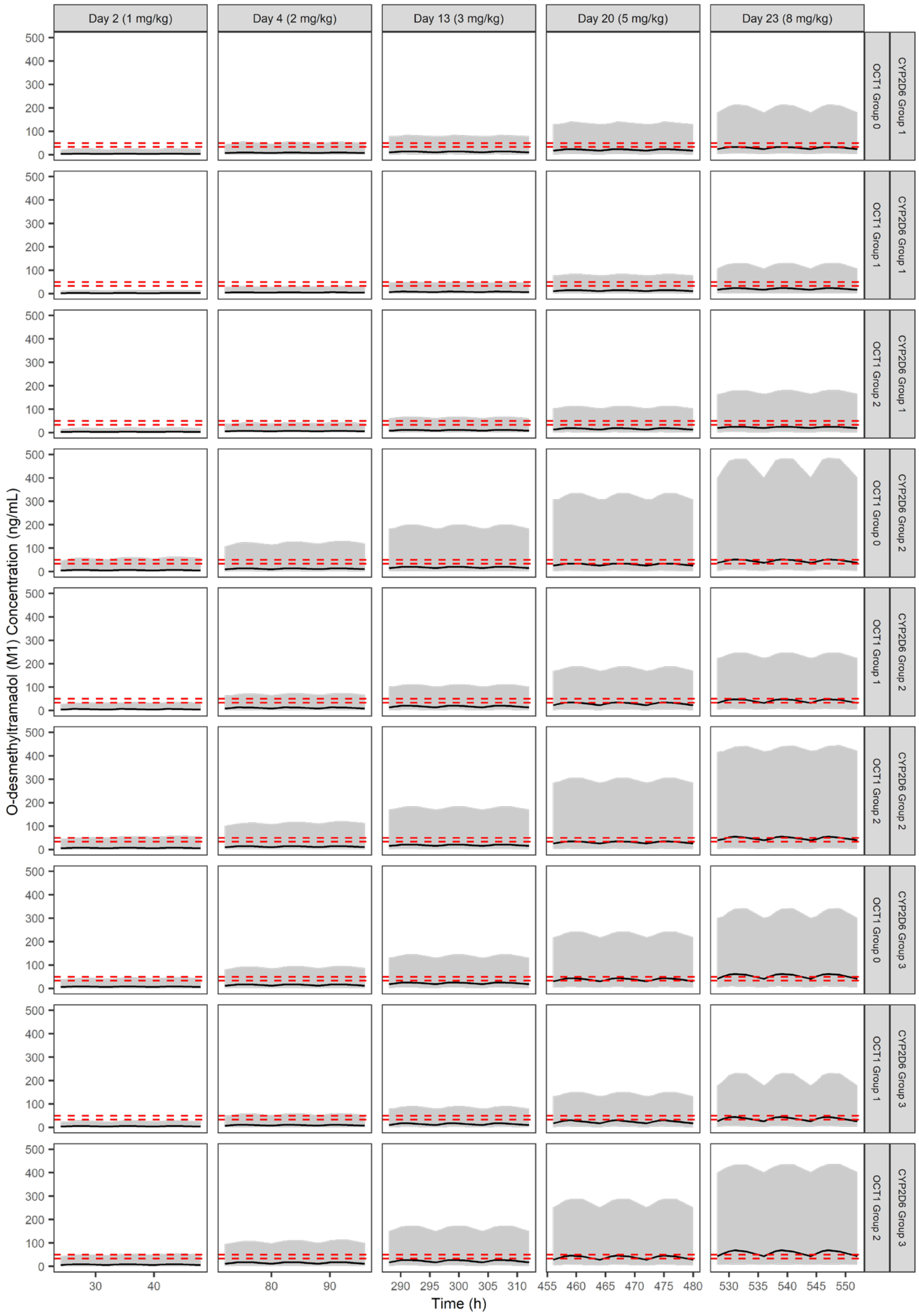

Figure S8. Predicted O-desmethyltramadol concentration vs time profiles for different genotye/phentotype groupings following each titration step with oral tramadol. . In each panel, the solid lines represent the median of the simulated profiles along with the corresponding 95% prediction intervals depicted in the shaded areas. The red dotted lines display M1's putative efficacious concentration window for analgesia (n=900; PMA = 51-976; weight = 4.7-73.4). See Table 2 in the main manuscript for the criteria used for the different groups used as discrete covariates for modelling and simulation purposes.

TABLE S1. Summary statistics for tramadol (upper panel) and O-desmethyltramadol (M1) (lower panel). AUC, Css and Cmax are shown for patients with polymorphism combinations of clinical concern (CYP2D6-G3/OCT1-G0 and CYP2D6-G2/OCT1-G0)* following stratification according to a titration schedule including oral doses of 1,2,3,5, 8 mg/kg tramadol. Values are medians and 90%⁻ prediction intervals. See Figure 4 for the time course of concentrations of tramadol and its metabolite M1 in these subgroups.

Tramadol

Parameter	Tramadol dose (mg/kg/day)	CYP2D6-G3+OCT1-G0	CYP2D6-G2+ OCT1-G0
AUC (ng*h/mL)	1	1335.63 (507.5-3235.72)	1432.91 (538.41-3509.42)
	2	2672.4 (1014.99-6559.86)	2869.42 (1076.85-7161.9)
	3	4013.92 (1522.5-9996.73)	4309.86 (1615.32-10967.83)
	5	6689.85 (2537.5-16661.28)	7183.06 (2692.18-18279.85)
	8	9736.38 (3778.51-24020.68)	10498.13 (4028.1-26423.09)
Css (ng/mL)	1	55.65 (21.15-134.82)	59.7 (22.43-146.23)
	2	111.35 (42.29-273.33)	119.56 (44.87-298.41)
	3	167.25 (63.44-416.53)	179.58 (67.3-456.99)
	5	278.74 (105.73-694.22)	299.29 (112.17-761.66)
	8	405.68 (157.44-1000.86)	437.42 (167.84-1100.96)
Cmax (ng/mL)	1	82.01 (41.42-167.86)	85.71 (42.94-180.25)
	2	164.03 (82.84-337.38)	171.48 (85.89-363.29)
	3	246.07 (124.27-508.24)	257.29 (128.83-549.42)
	5	410.12 (207.11-847.08)	428.81 (214.72-915.7)
	8	597.72 (305.04-1228.41)	628.25 (317.24-1326.44)

O-desmethyltramadol

Parameter	Tramadol dose (mg/kg/day)	CYP2D6 G3+OCT1-G0	CYP2D6-G2+ OCT1 G0
AUC (ng*h/mL)	1	169.98 (21.15-1093.27)	138.28 (15.45-940.85)
	2	342.38 (42.44-2247.55)	277.17 (30.9-1960)
	3	517.21 (63.87-3459.28)	419.05 (46.36-3063.05)
	5	862.02 (106.45-5765.39)	698.41 (77.26-5105.09)
	8	1265.25 (138.36-7747.88)	1098.07 (123.62-7541.97)
Css (ng/mL)	1	7.08 (0.88-45.55)	5.76 (0.64-39.2)
	2	14.27 (1.77-93.65)	11.55 (1.29-81.67)
	3	21.55 (2.66-144.14)	17.46 (1.93-127.63)
	5	35.92 (4.44-240.22)	29.1 (3.22-212.71)
	8	52.72 (5.76-322.83)	45.75 (5.15-314.25)
Cmax (ng/mL)	1	8.05 (1.05-48.98)	6.65 (0.85-42.72)
	2	16.11 (2.09-98.68)	13.29 (1.7-87.06)
	3	24.18 (3.14-149.14)	19.94 (2.55-133.12)
	5	40.3 (5.24-248.55)	33.24 (4.25-221.88)
	8	61.67 (7.57-349.87)	51.79 (6.7-326.82)

***** See Table 2 in the main manuscript for the criteria used for the different groups used as discrete covariates for modelling and simulation purposes.

TABLE S2. Secondary pharmacokinetic parameters stratified by weight bands in paediatric patients with clinically relevant metabolic genotype/phenotype variants. Values are medians and 90%- prediction interval.

CYP2D6 Group 3 and OCT1 Group 0 - Tramadol

Dose (mg/kg)	Weight (Kg)	AUC (ng /mL*h)	Cmax (ng/mL)	Css (ng/mL)
1	<10	896.1<br>(369.36-2249.8)	61.1<br>(32.91-123.43)	37.3<br>(15.39-93.74)
	10-<20	1087.8<br>439.03-2547.59)	70.2<br>(36.68-137.28)	45.3<br>(18.29-106.15)
	20-<40	1257.0<br>(516.45-3046.44)	78.0<br>(42.31-159.44)	52.3<br>(21.52-126.93)
	≥40	1499.8<br>(599.01-3483.76)	89.6<br>(46.72-179.03)	62.4<br>(24.96-145.16)
2	<10	1792.3<br>(738.71-4512.89)	122.2<br>(65.82-246.99)	74.6<br>(30.78-188.04)
	10-<20	2175.8<br>(878.07-5122.84)	140.4<br>(73.36-274.88)	90.6<br>(36.59-213.45)
	20-<40	2516.2<br>(1032.9-6149.96)	156.1<br>(84.61-320.03)	104.8<br>(43.04-256.25)
	≥40	3005.9<br>(1198.07-7093.27)	179.3<br>(93.43-360.52)	125.2<br>(49.92-295.55)
3	<10	2688.6<br>(1108.07-6789.36)	183.4<br>(98.72-370.65)	112.0<br>(46.17-282.89)
	10-<20	3264.1<br>(1317.12-7728.31)	210.6<br>(110.04-412.79)	136.0<br>(54.88-322.01)
	20-<40	3776.4<br>(1549.35-9311.54)	234.1<br>(126.92-481.55)	157.3<br>(64.56-387.98)
	≥40	4517.9<br>(1797.17-10804.67)	269.1<br>(140.15-544.65)	188.2<br>(74.88-450.19)
5	<10	4480.9<br>(1846.77-11315.5)	305.6<br>(164.54-617.76)	186.7<br>(76.95-471.48)
	10-<20	5440.1<br>(2195.21-12880.69)	351.1<br>(183.4-687.98)	226.6<br>(91.47-536.7)
	20-<40	6294.0<br>(2582.26-15519.21)	390.3<br>(211.53-802.58)	262.2<br>(107.59-646.63)
	≥40	7529.9<br>(2995.3-18007.8)	448.5<br>(233.58-907.74)	313.7<br>(124.8-750.33)

8	<10	7169.6 (2954.88-18103.48)	489.0 (263.27-988.4)	298.7 (123.12-754.31)
	10-<20	8704.2 (3512.33-20604.63)	561.7 (293.44-1100.73)	362.6 (146.35-858.53)
	20-<40	10070.3 (4131.61-24818.06)	624.4 (338.45-1283.92)	419.6 (172.15-1034.09)
	≥40	10203.0 (4006.11-24738.56)	608.2 (308.84-1249.93)	425.1 (166.92-1030.77)

CYP2D6 Group 3 and OCT1 Group 0 – O-desmethyltramadol

Dose (mg/kg)	Weight (Kg)	AUC (ng /mL*h)	Cmax (ng/mL)	Css (ng/mL)
1	<10	154.9 (20.33-1085.23)	7.8 (1.11-48.9)	6.4 (0.85-45.22)
	10-<20	309.8 (40.66-2220.72)	15.7 (2.21-98.87)	12.9 (1.69-92.53)
	20-<40	464.7 (61-3361.65)	23.6 (3.32-149.69)	19.3 (2.54-140.07)
	≥40	774.5 (101.66-5602.7)	39.3 (5.54-249.48)	32.2 (4.24-233.45)
2	<10	1239.3 (162.65-8961.56)	62.9 (8.86-398.91)	51.6 (6.78-373.4)
	10-<20	142.2 (19.96-995.54)	7.3 (1.1-45.04)	5.9 (0.83-41.48)
	20-<40	284.5 (39.91-2010.56)	14.7 (2.19-90.33)	11.8 (1.66-83.77)
	≥40	426.8 (59.87-3045.14)	22.1 (3.29-135.86)	17.7 (2.49-126.88)
3	<10	711.3 (99.78-5075.32)	36.8 (5.49-226.43)	29.6 (4.16-211.47)
	10-<20	1138.2 (159.65-8117.69)	58.9 (8.78-362.25)	47.4 (6.65-338.24)
	20-<40	166.2 (21.5-1149.79)	8.1 (1.15-51.53)	6.9 (0.9-47.91)
	≥40	333.2 (42.99-2344.78)	16.2 (2.31-103.92)	13.8 (1.79-97.7)

5	<10	501.7 (64.5-3590.21)	24.3 (3.47-156.29)	20.9 (2.69-149.59)
	10-<20	836.2 (107.49-5983.76)	40.5 (5.79-260.49)	34.8 (4.48-249.32)
	20-<40	1338.0 (171.99-9564.48)	64.8 (9.27-416.75)	55.7 (7.17-398.52)
	≥40	184.9 (24.91-1309.22)	8.7 (1.27-59.54)	7.7 (1.04-54.55)
8	<10	371.7 (50.25-2751.86)	17.6 (2.54-121.67)	15.4 (2.09-114.66)
	10-<20	561.8 (75.42-4322.54)	26.4 (3.81-186.51)	23.4 (3.14-180.11)
	20-<40	936.4 (125.71-7204.15)	44.0 (6.35-310.84)	39.0 (5.24-300.17)
	≥40	1284.4 (171.73-9867.96)	60.6 (8.59-423.93)	53.5 (7.16-411.16)

CYP2D6 Group 2 and OCT1 Group 0 – Tramadol

**Dose (mg/kg)**	**Weight (Kg)**	**AUC (ng /mL*h)**	**Cmax (ng/mL)**	**Css (ng/mL)**
1	<10	993.5 (371.93-2416.38)	65.4 (34.4-131.77)	41.4 (15.5-100.68)
	10-<20	1173.3 (461.24-2733.82)	73.3 (38.3-144.42)	48.8 (19.22-113.91)
	20-<40	1372.9 (530.98-3237.19)	82.9 (42.46-167.08)	57.2 (22.12-134.88)
	≥40	1622.0 (632.92-3831.3)	94.3 (48.69-194.8)	67.5 (26.37-159.64)
2	<10	1987.1 (743.87-4846.85)	130.8 (68.79-263.89)	82.8 (30.99-201.95)
	10-<20	2348.8 (922.48-5517.32)	146.6 (76.6-289.82)	97.8 (38.44-229.89)
	20-<40	2748.3 (1062.08-6557.78)	165.8 (84.91-335.92)	114.5 (44.25-273.24)
	≥40	3252.6 (1265.9-7846.06)	188.8 (97.39-393.84)	135.5 (52.75-326.92)

3	<10	2980.9 (1115.82-7291.43)	196.2 (103.2-396.36)	124.2 (46.49-303.81)
	10-<20	3526.7 (1383.74-8355.74)	219.9 (114.9-436.22)	146.9 (57.66-348.16)
	20-<40	4125.9 (1593.3-9951.65)	248.7 (127.37-506.52)	171.9 (66.39-414.65)
	≥40	4889.8 (1898.93-12067.67)	283.3 (146.08-597.15)	203.7 (79.12-502.82)
5	<10	4968.3 (1859.7-12152.48)	327.0 (171.99-660.6)	207.0 (77.49-506.35)
	10-<20	5877.8 (2306.22-13926.29)	366.6 (191.5-727.03)	244.9 (96.09-580.26)
	20-<40	6876.5 (2655.49-16586.07)	414.5 (212.28-844.2)	286.5 (110.65-691.09)
	≥40	8149.6 (3164.86-20112.87)	472.2 (243.46-995.24)	339.5 (131.87-838.04)
8	<10	7949.2 (2975.48-19442.46)	523.3 (275.17-1056.87)	331.2 (123.98-810.1)
	10-<20	9404.3 (3689.92-22268.79)	586.6 (306.4-1163.02)	391.8 (153.75-927.87)
	20-<40	11002.2 (4248.83-26516.28)	663.3 (339.64-1350.21)	458.4 (177.03-1104.85)
	≥40	10978.6 (4288.75-27527.84)	642.9 (320.77-1368.32)	457.4 (178.7-1146.99)

CYP2D6 Group 2 and OCT1 Group 0 – O-desmethyltramadol

**Dose (mg/kg)**	**Weight (Kg)**	**AUC (ng /mL*h)**	**Cmax (ng/mL)**	**Css (ng/mL)**
1	<10	123.3 (14.96-1078.56)	6.2 (0.8-48.57)	5.1 (0.62-44.94)
	10-<20	247.6 (29.92-2187.03)	12.4 (1.6-97.41)	10.3 (1.25-91.13)
	20-<40	371.8 (44.88-3347.81)	18.6 (2.4-146.52)	15.4 (1.87-139.49)
	≥40	619.7 (74.79-5579.65)	31.1 (4-244.19)	25.8 (3.12-232.49)

2	<10	991.5 (119.67-8905.64)	49.7 (6.39-390.67)	41.3 (4.99-371.07)
	10-<20	118.0 (14.38-946.9)	5.9 (0.75-42.3)	4.9 (0.6-39.45)
	20-<40	236.2 (28.76-1921.71)	11.9 (1.49-84.9)	9.8 (1.2-80.07)
	≥40	354.7 (43.25-2953.85)	17.8 (2.24-129)	14.7 (1.8-123.08)
3	<10	591.2 (72.09-4923.12)	29.7 (3.74-215)	24.6 (3-205.13)
	10-<20	946.0 (115.32-7870.21)	47.6 (5.98-343.3)	39.4 (4.81-327.93)
	20-<40	138.9 (16.55-1051.19)	6.7 (0.83-47.57)	5.7 (0.69-43.8)
	≥40	279.5 (33.1-2154.3)	13.4 (1.66-95.98)	11.6 (1.38-89.76)
5	<10	420.0 (49.7-3351.6)	20.2 (2.49-146.42)	17.5 (2.07-139.65)
	10-<20	700.0 (82.83-5585.92)	33.7 (4.16-244.03)	29.1 (3.45-232.75)
	20-<40	1120.0 (132.52-8922.56)	54.0 (6.65-390.07)	46.6 (5.52-371.77)
	≥40	158.9 (19.55-1104.78)	7.5 (0.98-50.05)	6.6 (0.81-46.03)
8	<10	320.6 (39.15-2290.21)	15.1 (1.97-101.59)	13.3 (1.63-95.43)
	10-<20	484.9 (59.82-3606.94)	22.6 (2.96-155.19)	20.2 (2.49-150.29)
	20-<40	808.2 (99.69-6011)	37.8 (4.93-258.66)	33.6 (4.15-250.46)
	≥40	1102.8 (134.46-8275.31)	51.5 (6.6-359.19)	45.9 (5.6-344.8)

See Table 2 in the main manuscript for the criteria used for the different groups used as discrete covariates for modelling and simulation purposes.

Main limitations

We acknowledge a number of limitations in our analysis. First, the limited number of patients with genotyping data has required us to use prior distributions and fixing the value of some parameters, based on previous estimates, such as VM1, which was set to the value reported by Allegaert et al [1]. Unfortunately, the lack of experimental data on M1, has not allowed estimation of the true formation clearance. Indeed, M1 plasma concentration may be the most important factor for analgesia as it's M1 that is primarily responsible for the opioid-related analgesia of tramadol. Essentially, if one were to dose a patient with tramadol without knowing the genotype status an unknown dose of M1 is being given. However, a recent study in adults [2] gives an interesting perspective in considering the isolated metabolite as an analgesic drug, as opposed to tramadol (the "prodrug") in order to circumvent the metabolic complications, we have faced during the estimation of the covariate effects associated with genetic polymorphisms. Pharmacokinetic data from a new study investigating intravenous M1 would help enormously in reducing the uncertainty regarding its contribution the overall analgesic response.

Another important, but yet less obvious and potentially hazardous issue are drug-drug interactions. We have not considered scenarios in which pharmacokinetic interactions lead to metabolic inhibition. One situation that could have direct clinical implications, is the co-administration of SNRI (serotonin-norepinephrine reuptake inhibitor) to subjects who are poor metabolisers. The much lower conversion of tramadol to M1 in this instance may cause a drug-drug interaction leading to serotonin toxicity, which has also been seen with other opioids [3].

From a modelling perspective, it should be noted that the criteria used to parameterise the effect of genetic variants and cluster the subjects based on the activity scores was such that one could disentangle the genetic effect from maturational processes, i.e., making it independent from post-natal or post-menstrual age. As indicated above for CYP2D6, our working assumption included a separate term for maturational processes. Whilst the maturation of CYP450 isozymes has been widely investigated, understanding of age-related changes in OCT1 is rather limited. Previous investigations by Hahn and colleagues [4] have shown an age-dependent increase in OCT1 expression from birth up to 8 to 12 years old, and suggested that maturation may be complete at 7 years after birth. However, there is insufficient data to establish the relationship between expression levels, OCT1 functional capacity and its effect upon the disposition of OCT1 substrates in neonates and small infants. Therefore, it was assumed that changes in OCT1 expression across the age range included in the analysis had minor clinical implication.

Lastly, our analysis has relied on a target therapeutic range for tramadol, without factoring the presence of varying levels of M1. Clearly, this does not invalidate the proposed range, but restricts it to the patient population whose genotype/phenotype is not associated with ultra-rapid metabolism. The implications of the UM phenotype seem to persist across the whole paediatric population, irrespective of age or body weight.

Despite the limitations, our simulation results suggest the need for slow, stepwise titration to effect and capping of the maximum dose of tramadol to be used in children with body weight < 40 kg at 5 mg/kg up to a maximum of 400 mg. This should mitigate the heightened response from UR patients while allowing

for increased dosing if insufficient analgesia is seen after the initial titration steps (in the case of PMs). In any case, greater alertness to reduced effectiveness is also recommended, irrespective of the availability of genotyping data indicating poor metabolism [5]. On the other hand, it may be difficult to predict the influence of decreased CYP2D6 metabolism on the overall analgesic effects, because the increase in the tramadol to O-desmethyltramadol ratio may result in enhanced SNRI activity of tramadol. In theory, this effect could counteract some of the lost O-desmethyltramadol opioid receptor-mediated analgesic effects. In another study [6], a dose increase of 50% and reduction of 25% is recommended for patients with IM and UM phenotypes, respectively. This adjustment aims to ensure plasma exposure to M1 is comparable to levels observed in EM patients. However, for the reasons previously mentioned, we believe that a lower starting dose, with a stepwise titration to response is the most appropriate approach for the paediatric population.

References:

1. Allegaert K, Anderson BJ, Verbesselt R, Debeer A, de Hoon J, Devlieger H, Van Den Anker JN, Tibboel D. Tramadol disposition in the very young: an attempt to assess *in vivo* cytochrome P-450 2D6 activity. Br J Anaesth. 2005; 95(2):231-9.

2. Zebala JA, Searle SL, Webster LR, Johnson MS, Schuler AD, Maeda DY, Kahn SJ. Desmethyl-tramadol has the safety and analgesic profile of tramadol without its metabolic liabilities: Consecutive randomized, double-blind, placebo- and active comparator-controlled trials. J Pain. 2019; 20(10):1218-1235.

3. Kesavan S, Sobala GM. Serotonin syndrome with fluoxetine plus tramadol. J R Soc Med. 1999; 92(9):474-5.

4. Hahn D, Emoto C, Vinks AA, Fukuda T. Developmental changes in hepatic organic cation transporter OCT1 protein expression from neonates to children. Drug Metab Dispos. 2017;45(1):23-26.

5. Rollinson V, Turner R, Pirmohamed M. Pharmacogenomics for primary care: an overview. Genes (Basel). 2020; 11(11):1337.

6. Xu M, Zheng L, Zeng J, Xu W, Jiang X, Wang L. Physiologically based pharmacokinetic modeling of tramadol to inform dose adjustment and drug-drug interactions according to CYP2D6 phenotypes. Pharmacotherapy 2021; 41(3):277-290.

Control stream file: Final model

```
$PROBLEM    TRAMADOL

;; 1. Based on: Tramadol_only_FINAL_ETAs_ERROR_M1_QM_new

;; 2. Description: TRAMADOL Proportional & additional error model

;; x1. Author: Paul


$INPUT      ID       TIME     RATE     AMT    DUR     MDV    CMT    DV    PNA    PMA    WT    SEX
            STUDY    CYP2D6   CYP2DI   OCT    OCTDI   CYP




$DATA    neo_oct_cyp_NEWgroups.csv IGNORE=@ IGNORE=(CYP.EQ.0) IGNORE=(ID.EQ.69) IGNORE=(ID.EQ.101) IGNORE=(ID.EQ.76)
IGNORE=(ID.EQ.75)IGNORE=(ID.EQ.66)

;IGNORE=(ID.EQ.2)

;IGNORE=(ID.EQ.96)  IGNORE=(ID.EQ.56) IGNORE=(ID.EQ.4)

;IGNORE=(ID.EQ.95)

;IGNORE=(ID.EQ.10)

;IGNORE=(ID.EQ.84)

;IGNORE=(ID.EQ.67) IGNORE=(ID.EQ.50)


;$SUBROUTINE ADVAN6 TOL=3

$SUBROUTINE ADVAN6 TOL=3

$MODEL      COMP=(CENT) COMP=(PER) COMP=(MCENT) COMP=(MPER)


$PK

IF (CYP.EQ.1) CLPP = THETA(1)

IF (CYP.EQ.2) CLPP = THETA(2)

IF (CYP.EQ.3) CLPP = THETA(3)


TVCLPO = THETA(4) *((WT/70)**0.75)*(1/(1+(((PMA)/39.1)**(-6.76))))          ; Units - L/h (70kg) CL OF T BY OTHER PATHWAYS

CLPO    = TVCLPO*EXP(ETA(1))


TVQP    = THETA(5)*((WT/70)**0.75)

QP      = TVQP


TVVP1   = THETA(6)*((WT/70)**1)                                          ; Units - L (70kg)
```

```
VP1     = TVVP1*EXP(ETA(2))

TVVP2   = THETA(7)*((WT/70)**1)                                              ; Units - L (70kg)
VP2     = TVVP2                                              ; Units - L (70kg)

IF (CYP.EQ.1) TVCLPM=THETA(8)*((WT/70)**0.75)*(1/(1+(((PMA)/39.8)**(-9))))                ; Units - L/h (70kg)
IF (CYP.EQ.2) TVCLPM=THETA(9)*((WT/70)**0.75)*(1/(1+(((PMA)/39.8)**(-9))))                ; Units - L/h (70kg)
IF (CYP.EQ.3) TVCLPM=THETA(10)*((WT/70)**0.75)*(1/(1+(((PMA)/39.8)**(-9))))               ; Units - L/h (70kg)

IF (CYP.EQ.1) CLPM = TVCLPM*EXP(ETA(3))
IF (CYP.EQ.2) CLPM = TVCLPM*EXP(ETA(4))
IF (CYP.EQ.3) CLPM = TVCLPM*EXP(ETA(5))

IF (OCT.EQ.0) TVCLMO=THETA(11) *((WT/70)**0.75)*(1/(1+(((PMA)/47.7)**(-3.4))))
IF (OCT.EQ.1) TVCLMO=THETA(12) *((WT/70)**0.75)*(1/(1+(((PMA)/47.7)**(-3.4))))
IF (OCT.EQ.2) TVCLMO=THETA(13) *((WT/70)**0.75)*(1/(1+(((PMA)/47.7)**(-3.4))))

IF (OCT.EQ.0) CLMO    = TVCLMO;*EXP(ETA(6))
IF (OCT.EQ.1) CLMO    = TVCLMO;*EXP(ETA(7))
IF (OCT.EQ.2) CLMO    = TVCLMO;*EXP(ETA(8))

TVQM    = THETA(14)*((WT/70)**0.75)                                          ; Units - L/h (70kg)
QM      = TVQM *EXP(ETA(6))

TVVM1   = THETA(15)*((WT/70)**1)                                             ; Units - L (70kg)
VM1     = TVVM1*EXP(ETA(7))

TVVM2   = THETA(16)*((WT/70)**1)                                             ; Units - L (70kg)
VM2     = TVVM2

CLPP = CLPO + CLPM

S1=VP1
S2=VP2
S3=VM1
```

```
S4=VM2

$DES
DADT(1) = (QP/VP2)*A(2)-(QP/VP1)*A(1)-(CLPO/VP1)*A(1)-(CLPM/VP1)*A(1)                    ; Central Compartment
DADT(2) = (QP/VP1)*A(1)-(QP/VP2)*A(2)                                                    ; Peripheral Compartment
DADT(3) = (CLPM/VP1)*A(1)-(QM/VM1)*A(3)*0.947+(QM/VM2)*A(4)-(CLMO/VM1)*A(3)*0.947        ; M1 Compartment
DADT(4) = (QM/VM1)*A(3)*0.947-(QM/VM2)*A(4)                                              ; M1 Peripheral Compartment

$ERROR
ITER = IREP
IPRED=F

IF (CMT.EQ.1) Y = F*(1+EPS(1))+EPS(2)
IF (CMT.EQ.3) Y = F*(1+EPS(3))+EPS(4)
IRES = DV-IPRED

$THETA
(28.7)  FIX   ; CLPP [CYP]       [1]
(36.3)  FIX   ; CLPP [CYP]       [2]
(43)    FIX   ; CLPP [CYP]       [3]
(0, 30)       ; CLP0 [CYP]       [4]
(0, 111)  FIX ; QP   [OCT]       [7]
(0, 141)  FIX ; VP1  [OCT]       [8]
(0, 102)  FIX ; VP2  [OCT]       [9]
(0,5)         ; CLPM             [10]
(0,10)        ; CLPM             [11]
(0,12)        ; CLPM             [12]
(0,40)        ; CLMO [CYP]1      [13]
(0,40)        ; CLMO [CYP]1      [14]
(0,40)        ; CLMO [CYP]1      [15]
(635)     FIX ; QM               [16]
(78.9)    FIX ; VM1              [17]
(0,100)       ; VM2              [18]

$OMEGA
```

```
0.1        ;   CLPO [1]

0.211    FIX ;   VP1  [4]

0.1        ;   CLPM [5]

0.1        ;   CLPM [6]

0.1        ;   CLPM [7]

1.98     FIX ;   QM   [8]

1.45     FIX ;   VM1  [9]


$SIGMA

0.0159   FIX ; CVCP

0.00001  FIX ; CVCM

0.1;

0.1;


$ESTIMATION METHOD=1 INTER MAXEVAL=99999 SIG=3 PRINT=1 NOABORT POSTHOC

$COV

$TABLE ID WT AMT TIME PMA CMT DV MDV IPRED CWRES CYP2D6 CYP2DI CLPO CLPM CLMO OCT PRED CYP ITER

NOAPPEND ONEHEADER NOPRINT FILE=Tramadol_FINAL_2023.csv
```
